# Study of the β-oxygen effect in the Barton–McCombie reaction for the total synthesis of (4*R*,5*R*)-4-hydroxy-γ-decalactone (Japanese orange fly lactone): a carbohydrate based approach†

**DOI:** 10.1039/d2ra04531a

**Published:** 2022-09-07

**Authors:** Janardana Reddi Desireddi, Mora Mallikarjuna Rao, Kiran Kumar Murahari, Rajashekar Reddy Nimmareddy, Thirupathi Mothe, Arun Kumar Lingala, Bhimcharan Maiti, Ravinder Manchal

**Affiliations:** Aragen life sciences Private Limited (formerly known as GVK Biosciences Private Limited), Medicinal Chemistry Division 28A, IDA Nacharam Hyderabad-500076 Telangana India janardanareddi@gmail.com; Department of Chemistry Chaitanya (Deemed to be University) Warangal-506001 Telangana India ravinder@chaitanya.edu.in; Accrete Pharmaceuticals Private Limited Tangadpalli Village, Choutuppal Mandal, Yadadri Bhuvanagiri District-508252 Telangana India

## Abstract

Efficient and facile synthesis of Japanese orange fly lactone (1) was achieved from a commercially available d-glucose by investigating the Barton–McCombie reaction with furanose anomeric isomers (**12*α***, **β**) with an overall yield of 12.6%. During the course of this synthesis, the β-oxygen effect was discovered in the deoxygenation step at the C-3 position using the Barton–McCombie reaction, where the substrate allows the effect to operate in one of the isomers but not in the other. Under the same reaction conditions, xanthate derived from the β-furanose isomer affords a high yield of deoxygenated product, whereas the α-isomer produces a very low yield. The key transformations used were Wittig olefination, TEMPO mediated oxidation, and Barton–McCombie deoxygenation, resulting in a concise total synthesis of Japanese orange fly lactone (1). Our success will allow for further biological studies of this natural product, as well as opportunities for developing new potentially promising pheromones.

## Abstract 2

The Japanese orange fly lactone was synthesised with a chiral pool approach using commercially available, starting material d-glucose. This synthesis encountered a dominant structurally functioning impact in the Barton–McCombie reaction known as the β-oxygen effect.

## Graphical abstract

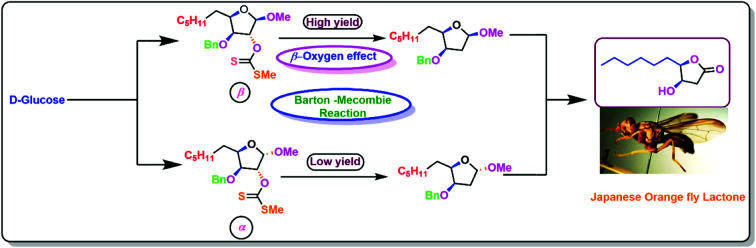

## Introduction

**Fig. 1:**
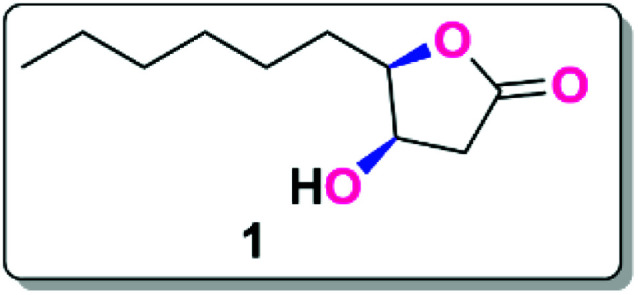
Structure of Japanese orange fly lactone.

The substances which mediate communication between organisms are known as semiochemicals and these are classified into pheromones and allelochemicals. Pheromones are of exocrine origin and are volatile compounds secreted by animals into the external environment which brings about specific communication among the same species. Pheromones were isolated and identified by German chemists Karlson and Butenandt in 1959. The activity of semiochemicals in insects is to help in the identification of food sources, the location of mates and hosts for oviposition and protection from predators (Fig. 1).^1^

Chemical communication is an essential and important role in insect survival and it plays a significant part in how they adapt their behaviour to the local environment. Presently pheromones and semiochemicals are in wide use to manage agricultural, stored products, mass trapping, mating disruption, attract, kill and push–pull strategies. The actual existence of pheromones has been known for centuries. Because of the improper use of pesticides has led to issues including resurgence, insecticide resistance, secondary pest outbreaks, death of non-target species and environmental contamination, the use of semiochemicals in agriculture has expanded.^2^

Chirality frequently has a significant impact on the biological activity of molecules. This phenomenon is crucial for the pheromone that insects make.^3^ Many different physiologically active compounds include lactone motif. The synthesis of chiral lactones continues to be a difficult topic in organic synthesis.^4^ Insect pheromones, antifungal agents, flavourings and plant essential oils are just a few examples of natural compounds that include the lactone moiety. Lactone semiochemicals are produced by insects, animals and bacteria, these creatures impact our environment through chemical interactions. This has been noticed mainly in insects, on which the majority of research has been focused due to their economic importance. The lactone motif is a structural feature found in all currently known compounds, such as those found in bacteria or insects.^5^

*γ*-Butyrolactones are an important family of chemicals because they can be readily transformed into butenolides, furans, cyclopentenones and other compounds. Recently, there has been an increase interest in finding synthetic approaches to poly modified *γ*-butyrolactones and various physiologically active natural compounds with the skeleton of *γ*-butyrolactones as a prominent structural characteristic.^6,7^

In this article, we'd like to present the Bactrocera tsunami (Miyake), also known as the Japanese orange fly. It is one of the most significant pests of commercial citrus in Japan and has a limited distribution in China and Vietnam, but it has the capacity to colonise countries outside of Asia. Since 1947, there have been significant problems with commercial citrus plantations in China and Japan, where 60% of the fruits were destroyed and 50% of the oranges at Kiangtsin in the Szechwan Province in southern China during 1940. Additionally, the United States is not much affected.

In the present investigation we made an attempt to synthesize the isolated product of lactone which is the key source of the insect Japanese orange fly. For the first time this lactone (4*R*,5*R*) was isolated by Ono and co-workers. The primary ingredient of the well-characterized ethanolic extract of tissue from the male rectal glands of the Japanese orange fly. This lactone is present in female and immature males in a lesser quantity, which indicates that the presence of (4*R*,5*R*)-4-hydroxy-*γ*-decalactone is responsible for the reproductive behaviour in the mature males.

**Scheme 1:**
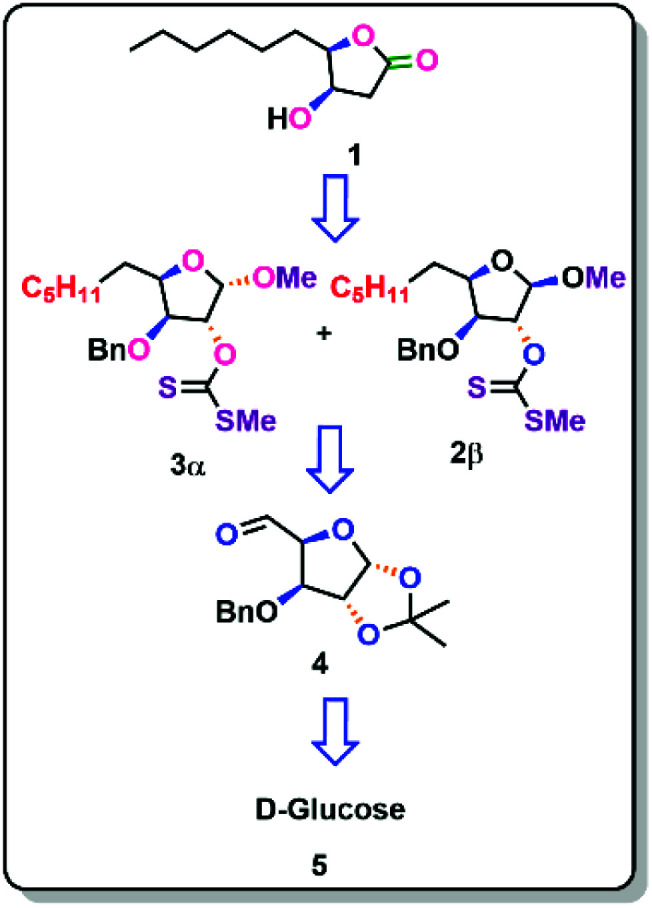
Retrosynthetic strategy of Japanese orange fly lactone (1).

The (4*R*,5*R*)-4-hydroxy-*γ*-decalactone skeleton is a *γ* -lactone with a hydroxyl group at the C-4 position of the ring and a six-carbon saturated chain at the C-5 position. We were drawn to the synthesis of the Japanese orange fly lactone (1) because of its structural properties and biological activity over pest control in agricultural applications. According to the retrosynthetic scheme of the Japanese orange fly lactone (1), it might be produced from the deoxygenation of the xanthate *via* the Barton–McCombie reaction, which could be acquired from the alcohol product, which can be conveniently accessible from d-glucose (Scheme 1).

## Results and discussions

Chiral pool synthesis is a useful method for synthesising enantiopure organic molecules from readily available enantiomerically pure compounds because of its low cost, abundance, and general renew ability. Some of the earlier approaches to the synthesis of *γ*-lactones, such as (+)-muricatacin, 3,4-disubstituted *γ*-lactones, 2-alken-4-olides and 3-hydroxydecano-4-lactones proceeds through a dihydroxylation and lactonization pathway. The chiral pool technique is used in our current study, using d-glucose as the starting material, followed by Barton–McCombie deoxygenation, demethylation, and lactonization.^8^

Using acetone and CuSO_4_ in the presence of H_2_SO_4_, d-glucose was protected as di acetonide (6) in an 86% yield.^9^ The free hydroxyl group at the C-4 position was then benzylated with benzyl bromide and NaH to obtain benzyl acetonide (7) in 86% yield.^10^ The more acid-labile acetonide at C-6 and C-7 positions was cleaved with 60% AcOH to produce the diol (8) in 88% yield.^11^ The aldehyde (4)^12^ was obtained *via* oxidative cleavage of a diol with silica-supported NaIO_4_, which was then Wittig olefinated with pentyl triphenyl phosphoryl bromide in the presence of *n*-BuLi to generate the olefin (10) in 87% yield (across two processes) as E-isomer.^13^ The olefin was reduced with Pd/C in 93% yield to obtain the saturated derivative (11).^14^ After being treated with Amberlite IR-120 H^+^ in methanol, the saturated derivative yielded 86% of a mixture of methyl glycosides (12).^15^ The anomers formed in the above step was separated by Biotage chromatography and isolated both the anomeric compounds 12*α* (42%) & 12β (44%) and been characterized with the support of 2D NMR spectroscopic studies.

**Scheme 2:**
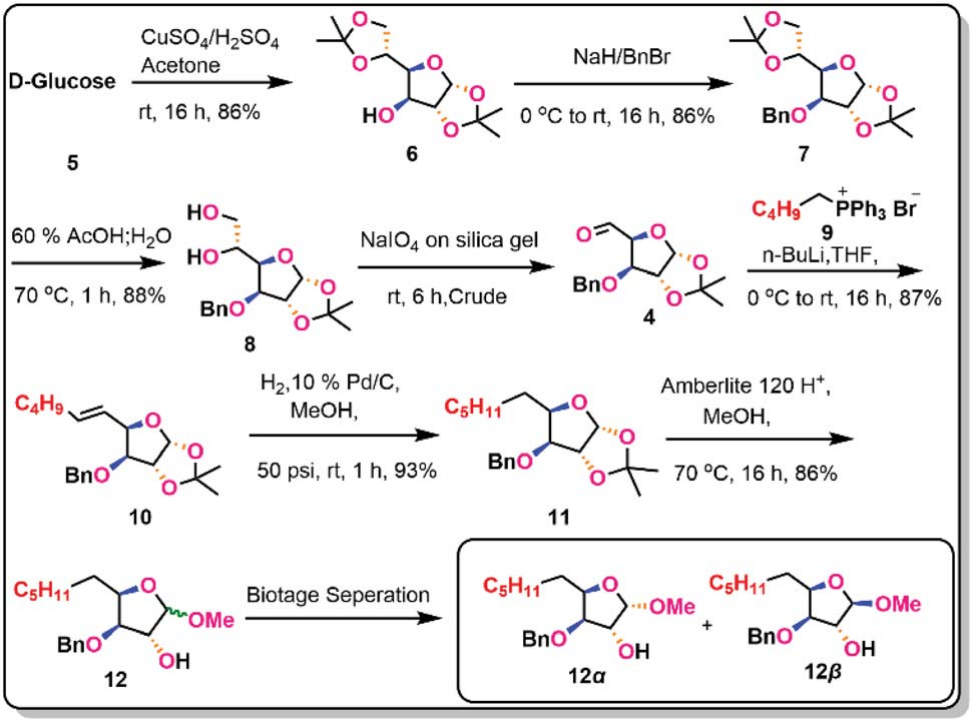
Synthesis of Anomeric isomers 12*α* and 12β.

In the depiction of the configuration of anomeric protons, one-dimensional ^1^H NMR offers a unique source of significant structural information on sugars. We investigated the ^1^H NMR for NOESY investigations to confirm the *α* and β-isomers of alcohol (12). Anomeric proton signals often emerge in the range of 4.3 to 5.9 ppm, while *α*-glycoside protons typically resonate at 0.3–0.5 ppm downfield from those of the equivalent β-glycosides. Furthermore, the *α*-anomeric proton resonates higher downfield (5.1 ppm) than the β-anomeric proton (4.5 ppm), distinguishing these two anomers by ^1^H NMR even at low field. Based on the chemical shift values and spin–spin coupling constants, the ^1^H NMR spectrum also provides information on the constituent's carbohydrates (*J*-values). The *α*-isomer has a greater coupling constant of (4.8 Hz) than the β-isomer (2.0 Hz) and the ^13^C-NMR indicates the anomeric carbon of the *α*-isomer has a chemical shift value of 101.5 ppm, while the β-isomer has a chemical shift value of 109.3 ppm, which is higher than the *α*-isomer (Scheme 2).

Based on the information presented above, it was determined that the chiral centre at the anomeric position in 12*α* was confirmed as S-configuration and in 12β was confirmed as R-configuration.^16^

Our next objective was to deoxygenate at the C-3 position of individual isomers (12*α* & 12β), where the alcohols were transformed into its xanthate esters in 89% (3*α*) and 92% (2β) yield respectively by using NaH, MeI, and CS_2_.^17^

By employing its xanthate ester, both isomers were individually exposed to the Barton–McCombie reaction to get the deoxygenated products. Due to the β-oxygen effect, 2β is more yielded 89% (16) than 3*α* 21% (13). This evidence demonstrates that the β-oxygen effect in the Barton–McCombie reaction is crucial in obtaining xanthate deoxygenation, which is consistent with the findings of Piscil *et al.*^18^ The β-oxygen effect in the Barton–McCombie reaction was elucidated from the orbital theory with the available literature. This may be summarised as follows.

**Scheme 3:**
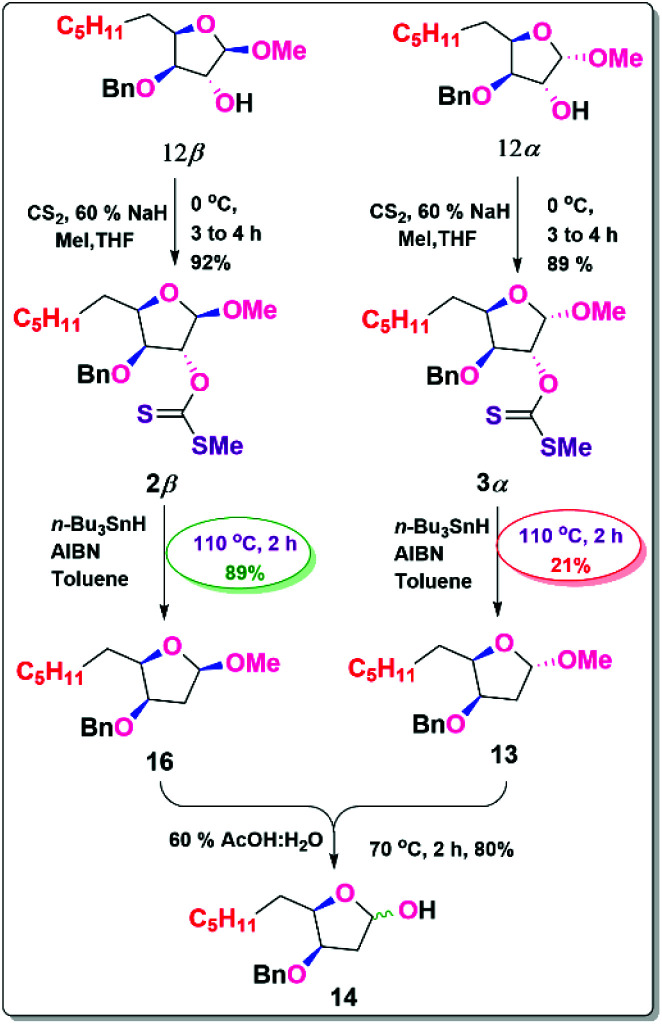
Synthesis of Lactol from 12β & 12α isomers.

A carbon-centred radical's stability and ease of production are both largely unaffected by the presence of a β-oxygen substituent.^19^ The stabilisation of carbon radicals is significantly influenced by β-bonded oxygen, which makes homolytic fission conceivable that otherwise would not be. The quantitative separation of the deoxygenation product suggests a favourable outcome in the Barton–McCombie reaction, probably as a result of the stereo electronic polar effect. Since this action is prevented by the *syn*-periplanar interaction between OMe and thiocarbonyl groups, the production of the equivalent deoxygenated product is far less advantageous (Scheme 3).^20^

**Scheme 4:**
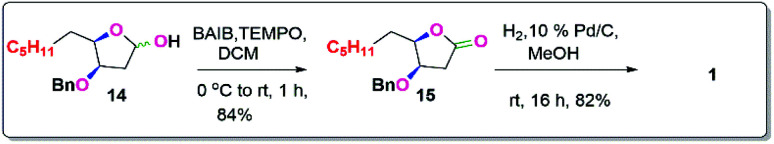
Synthesis of Japanese orange fly lactone (1).

The β-oxygen impact in the Barton–McCombie reaction is greatly favoured by unusual orbital interactions between the σ* orbital of the bond undergoing cleavage and C–O antibonding orbitals β-positioned hostile to the bond. Molecular orbital interactions are critical for inducing the β-scission of the alkoxy thiocarbonyl radical and thus delivering a deoxygenated product. The deoxygenation process through the β-scission is much faster in conformationally locked substrates, especially when the thiocarbonyl group is located synclinal to the β-oxygen atom.^21,22^ This research will be extremely beneficial in building a scalale procedure for β-deoxy furanose derivatives (Scheme 4).

After treatment with 60% AcOH: H_2_O, the deoxy methyl glycosides gave lactol (14) in 80% yield,^23^ which was then oxidised with BAIB/TEMPO to generate the benzyl derivative of Japanese orange fly lactone (15) in 84% yield.^24^ In methanol, the benzyl ether was cleaved with Pd/C to generate Japanese orange fly lactone (1) in 82% yield,^25^ which is consistent with analytical results from the isolated natural molecule.

## Experimental procedure

All compounds were acquired commercially and were utilised without additional purification. All reactions were carried out in oven-dried glassware with magnetic stirring (unless watery reagents were employed), and reactions involving air-sensitive compounds were carried out in an argon environment. Thin-layer chromatography was used to monitor all synthetic transformations (TLC). TLC was carried out using silica gel 60 F254 plates (aluminum plates). TLC spots are more noticeable in stains such as PMA and 5% H_2_SO_4_ in methanol. Purification of the crude chemicals produced was accomplished using flash column chromatography on silica gel using the Biotage equipment. Yields are spectroscopically pure, dried, and purified substances. ^1^H NMR spectra were recorded at 400 MHz, and ^13^C NMR at 100 MHz in CDCl_3_. Chemical shifts (*δ*) are reported in ppm and spectra were calibrated related to solvents residual proton chemical shifts (CDCl_3_, *δ* = 7.26) and solvents residual carbon chemical shifts (CDCl_3_, *δ* = 77.16) multiplicity is reported as follows: s = singlet, d = doublet, dd = doublet of the doublet, t = triplet, m = multiplet or unresolved and coupling constant *J* in Hz. Infrared spectra (IR) were recorded on a 0.1 mm KBr demountable cell. Optical rotations [*α*]_D_*T* were measured in CHCl_3_ with a digital polarimeter in a 2 mL and 5 mL cell of 1 diameter path length at 25 °C. High-resolution mass spectra (HRMS) were obtained by electrospray ionization or atmospheric pressure chemical ionization (ESI or APCI) using a Q-TOF mass spectrometer in positive ion mode (M + H or M + Na) as indicated.

### (3*aR*,5*S*,6*S*,6*aR*)-5-((*R*)-2,2-Dimethyl-1,3-dioxolan-4-yl)-2,2-dimethyltetrahydrofuro[2,3-*d*][1,3]dioxol-6-ol (6)

To a stirred solution of d-glucose 5 (50.0 g, 277.7 mmol, 1.0 eq.) in acetone (1.0 L) was added con. H_2_SO_4_ (1 mL) and stirred the contents at room temperature for 16 h. After completion of the reaction, the reaction mixture was neutralized with saturated NaHCO_3_ solution and concentrated the acetone under reduced pressure. The reaction mass was diluted with ethyl acetate (1 L), washed with water (200 mL), brine (200 mL), dried over anhydrous Na_2_SO_4_ and concentrated under reduced pressure to afford crude residue. The crude solid was stirred in hexane (250 mL) for 0.5 h and filtered the obtained solid to afford 6 (62 g, yield: 86%) as an off-white solid.*R*_f_ = 0.5 (50% EtOAc in hexane). ^1^H NMR (400 MHz, CDCl_3_): *δ* 5.95 (t, *J* = 3.6 Hz, 1H), 4.54 (d, *J* = 3.6 Hz, 1H), 4.37–4.32 (m, 2H), 4.19–4.15 (m, 1H), 4.09–4.06 (m, 1H), 2.51 (d, *J* = 3.6 Hz, 1H), 1.50 (s, 3H), 1.45 (s, 3H), 1.36 (s, 3H), 1.32 (s, 3H) ppm. MS (EI): *m*/*z* (M + H)^+^: 261.3.

### (3*aR*,5*R*,6*S*,6*aR*)-6-(Benzyloxy)-5-((*R*)-2,2-dimethyl-1,3-dioxolan-4-yl)-2,2-dimethyltetrahydrofuro[2,3-*d*][1,3]dioxole (7)

To a stirred solution of 6 (20 g, 76.92 mmol, 1.0 eq.) in anhydrous THF (200 mL) under argon atmosphere was added 60% NaH (6.15 g, 153.84 mmol, 2 eq.) at 0 °C over a period of 10 minutes and stirred the contents at same temperature for 1 h. Benzyl bromide (15.78 g, 73.83 mmol, 1.2 eq.) was added at 0 °C and stirred the contents at room temperature for 16 h. After completion of reaction, the reaction mixture was quenched with saturated aqueous ammonium chloride solution (300 mL) and extracted with ethyl acetate (500 mL). The organic layer was washed with water (150 mL), brine (150 mL), dried over anhydrous Na_2_SO_4_ and concentrated under reduced pressure to afford crude residue, which was purified by biotage chromatography using solvent gradient of 12% ethyl acetate in hexane to afford 7 (23.2 g, yield: 86%) as a pale brown oil. *R*_f_ = 0.4 (30% EtOAc in hexane). [*α*]^25^_D_ = −29.04° (*c* = 0.4% (W/V) in CHCl_3_).^1^H NMR (400 MHz, CDCl_3_): *δ* 7.35–7.29 (m, 5H), 5.90 (d, *J* = 3.6 Hz, 1H), 4.67–4.65 (m, 2H), 4.59–4.58 (m, 1H), 4.38–4.36 (m, 1H), 4.16–4.01 (m, 2H), 4.03–3.98 (m, 2H), 1.55–1.31 (m, 12H) ppm. IR (KBr) *ν*_max_ 3739, 3412, 2989, 2935, 2875, 2313, 1501, 1455, 1377, 1263, 1213, 1164, 1079, 1020, 947, 850, 750, 698, 639, 513 C m^−1^. MS (EI): *m*/*z* (M + Na)^+^: 373.5.

### (*R*)-1-((3*aR*,5*R*,6*S*,6*aR*)-6-(Benzyloxy)-2,2-dimethyltetrahydrofuro[2,3-*d*][1,3]dioxol-5-yl)ethane-1,2-diol (8)

To a stirred solution 7 (20.0 g, 57.14 mmol, 1.0 eq.) in 60% acetic acid : water (200 mL) was heated at 70 °C for 1 h. After completion of reaction, the reaction mixture was concentrated under reduced pressure to afford crude residue, which was purified by biotage chromatography using solvent gradient of 50% ethyl acetate in hexane to afford 8 (15.5 g, yield: 88%) as a pale-yellow oil. *R*_f_ = 0.3 (70% EtOAc in hexane). [*α*]^25^_D_ = −40.3° (*c* = 0.4% (w/v) in CHCl_3_). ^1^H NMR (400 MHz, CDCl_3_): *δ* 7.40–7.32 (m, 5H), 5.95 (d, *J* = 3.6 Hz, 1H), 4.76 (d, *J* = 12.0 Hz, 1H), 4.64 (d, *J* = 3.6 Hz, 1H), 4.59 (d, *J* = 12.0 Hz, 1H), 4.15–4.09 (m, 1H), 4.06–4.00 (m, 1H), 3.85–3.80 (m, 1H), 3.79–3.67 (m, 1H), 2.46–2.44 (d, *J* = 6.4 Hz, 1H), 1.99 (t, *J* = 6.0 Hz 1H), 1.49 (s, 3H), 1.33 (s, 3H) ppm. IR (KBr) *ν*_max_ 3739, 3427, 2988, 2935, 2875, 1739, 1642, 1456, 1377, 1264, 1213, 1164, 1077, 1020, 947, 850, 750, 698, 511 C m^−1.^MS (EI): *m*/*z* (M + Na)^+^: 333.2.

### (3*aR*,5*S*,6*S*,6*aR*)-6-(Benzyloxy)-2,2-dimethyltetrahydrofuro[2,3-*d*][1,3]dioxole-5-carbaldehyde (4)

To a stirred solution of 8 (15.0 g, 48.38 mmol, 1.0 eq.) in CH_2_Cl_2_ (300 mL) was added silica supported sodium periodate (98 g) and stirred the contents at room temperature for 6 h. After completion of reaction, the reaction mixture was filtered. The filtrate was dried over anhydrous Na_2_SO_4_ and concentrated under reduced pressure to afford crude aldehyde 4 (13.3 g, crude) as a colorless oil. The crude residue was directly used for the next step without further purification. *R*_f_ = 0.35 (70% EtOAc in hexane). IR (KBr) *ν*_max_ 3462, 2988, 2935, 1738, 1591, 1499, 1454, 1380, 1261, 1214, 1164, 1079, 1018, 854, 750, 697, 635, 434 C m^−1^.

### (3*aR*,5*R*,6*S*,6*aR*)-6-(Benzyloxy)-5-((*E*)-hex-1-en-1-yl)-2,2-dimethyltetrahydrofuro[2,3-*d*][1,3]dioxole (10)

To a stirred solution of bromo pentyl triphenylphosphorane 9 (59.13 g, 143.52 mmol, 3.0 eq.) in anhydrous THF (200 mL) under argon atmosphere was added *n*-BuLi (1.6 M in hexane) (74.7 mL, 119.6 mmol, 2.5 eq.) at 0 °C over a period of 15 minutes and stirred the contents at same temperature for 1 h. Crude aldehyde 4 (13.3 g, 47.84 mmol, 1.0 eq.) in anhydrous THF (70 mL) was added to above contents at 0 °C over a period of 0.5 h. The reaction was allowed to warm to room temperature and stirred for 16 h. After completion of reaction, the reaction mixture was quenched with saturated aqueous NH_4_Cl solution (250 mL) and extracted with Et_2_O (3 × 300 mL). The organic layer was washed with water (150 mL), brine (150 mL), dried over anhydrous Na_2_SO_4_ and concentrated under reduced pressure to afford crude residue, which was purified by biotage chromatography using solvent gradient of 5% ethyl acetate in hexane to afford 10 (13.9 g, yield: 87% over the two steps) as a pale-yellow oil. *R*_f_ = 0.3 (10% EtOAc in hexane). [*α*]^25^_D_ = −108.1° (*c* = 0.4% (w/v) in CHCl_3_). ^1^H NMR (400 MHz, CDCl_3_); *δ* 7.35–7.26 (m, 5H), 5.96 (d, *J* = 3.6 Hz, 1H), 5.74–5.64 (m, 2H), 4.96–4.93 (m, 1H), 4.66–4.62 (m, 2H), 4.57 (d, *J* = 12.4 Hz, 1H) 3.82 (d, *J* = 3.2 Hz, 1H), 2.16–2.03 (m, 2H), 1.52 (s, 3H), 1.58–1.26 (m, 7H), 0.87 (t, *J* = 2.4 Hz, 3H) ppm. ^13^C NMR (100 MHz, CDCl_3_): *δ* 137.6, 135.3, 128.4, 127.8, 127.5, 123.4, 111.3, 104.7, 83.3, 83.0, 75.9, 72.0, 31.6, 27.7, 26.81, 26.22, 22.2, 13.9 ppm. IR (KBr) *ν*_max_ 3741, 3420, 2991, 2925, 2861, 2314, 1648, 1554, 1508, 1457, 1377, 1267, 1213, 1164, 1079, 1021, 854, 804, 754, 698, 457 C m^−1.^ MS (EI): *m*/*z* (M + Na)^+^: 355.2.

### (3*aR*,5*R*,6*S*,6*aR*)-6-(Benzyloxy)-5-hexyl-2,2dimethyltetrahydrofuro[2,3-*d*][1,3]dioxole (11)

To a solution of 10 (18 g, 54.22 mmol, 1.0 eq.) in methanol (180 mL) under argon atmosphere was added 10% Pd/C (4.0 g) and degassed the reaction mass with 10 psi of hydrogen gas for 2 times, then hydrogenated the contents at 50 psi for 1 h. After completion of reaction the reaction mixture was filtered through Celite bed and the filtrate was concentrated under reduced pressure to afford crude residue, which was purified by biotage chromatography using solvent gradient of 4% ethyl acetate in hexane to afford 11 (16.8 g, yield: 93%) as a colorless oil. *R*_f_ = 0.35 (10% EtOAc in hexane). [*α*]^25^_D_ = −59.2° (*c* = 0.4% (w/v) in CHCl_3_). ^1^H NMR (500 MHz, CDCl_3_): *δ* 7.37–7.28 (m, 5H), 5.91 (d, *J* = 4.0 Hz, 1H), 4.72 (d, *J* = 12.0 Hz, 1H), 4.61 (d, *J* = 4.0 Hz, 1H), 4.49 (d, *J* = 12.4 Hz, 1H) 4.13–4.09 (m, 1H), 3.77 (d, *J* = 3.2 Hz, 1H), 1.76–1.65 (m, 2H), 1.58 (s, 3H), 1.49–1.18 (m, 11H), 0.88 (t, *J* = 6.8 Hz, 3H) ppm. ^13^C NMR (100 MHz, CDCl_3_): *δ* 137.6, 128.4, 127.8, 111.1, 104.6, 82.3, 81.8, 80.4, 71.7, 31.7, 29.4, 27.8, 26.7, 26.2, 26.0, 22.5, 14.0 ppm. IR (KBr) *ν*_max_ 3739, 3405, 2960, 2922, 2860, 2314, 1457, 1378, 1263, 1213, 1165, 1081, 1017, 948, 894, 854, 808, 753, 696, 632, 513, 462 C m^−1^. HRMS (ESI) calculated for C_22_H_34_0_4_ [M + NH_4_]^+^*m*/*z* = 352.2482, found: 352.2478.

### (2*S*,3*R*,4*R*,5*R*)-4-(Benzyloxy)-5-hexyl-2-methoxytetrahydrofuran-3-ol (12*α*) & (2*R*,3*R*,4*R*,5*R*)-4-(benzyloxy)-5-hexyl-2-methoxytetrahydrofuran-3-ol (12β)

To a stirred solution of 11 (16.0 g, 47.90 mmol, 1.0 eq.) in methanol (240 mL) under argon atmosphere was added Amberlite 120 H^+^ resin (4.8 g, 0.3 w/w) and refluxed the contents for 16 h. After completion of reaction, the reaction mixture was filtered and the filtrate was concentrated under reduced pressure. TLC shown the formation of 12*α* and beta 12β isomers with equal ratio (0.15*R*_f_ difference by TLC). These isomers were separated by biotage chromatography using solvent gradient of 6% ethyl acetate in hexane to afford 12*α* (6.2 g) as colorless oil and 12β as off white solid (6.4 g) (yield: 86%) (*R*_f_ = 0.3 & 0.45) (30% EtOAc in hexane).

### Analytical data of 12*α* isomer

[*α*]^25^_D_ = +48.60° (0.46% (w/v) in CHCl_3_). ^1^H NMR (400 MHz, CDCl_3_): *δ* 7.35–7.28 (m, 5H), 4.98–4.99 (d, *J* = 4.8 Hz, 1H), 4.75 (d, *J* = 12.0 Hz, 1H), 4.54 (d, *J* = 12.0 Hz, 1H), 4.25–4.22 (m, 1H), 4.12–4.09 (m, 1H) 3.81–3.79 (m, 1H), 3.48 (s, 3H), 2.85 (d, *J* = 6.0 Hz, 1H), 1.67–1.63 (m, 2H), 1.35–1.24 (m, 8H), 0.88 (t, *J* = 6.8 Hz, 3H) ppm. ^13^C NMR (100 MHz, CDCl_3_): *δ* 138.1, 128.3, 127.6, 101.5, 84.0, 78.9, 76.7, 71.4, 55.6, 31.8, 29.4, 28.9, 26.0, 22.6, 14.1 ppm.

IR (KBr) *ν*_max_ 3551, 3450, 3031, 2960, 2918, 2854, 1952, 1877, 1811, 1602, 1503, 1457, 1395, 1348, 1269, 1197, 1135, 1095, 991, 948, 896, 808, 753, 696, 650, 602, 538, 441 C m^−1^. MS (EI): *m*/*z* (M + Na) 359.19. HRMS (ESI): *m*/*z* calculated for [M + Na]^+^ = 331.1880, found: 331.1872.

### Analytical data of 12β isomer

[*α*]^25^_D_ = −55.5° (0.4% (w/v) in CHCl_3_). ^1^H NMR (400 MHz, CDCl_3_): *δ* 7.35 (d, *J* = 4.4 Hz 4H), 7.32–7.26 (m, 1H), 4.75 (d, *J* = 2.0 Hz, 1H), 4.68 (d, *J* = 12.4 Hz, 1H), 4.55 (d, *J* = 12.0 Hz, 1H), 4.22 (d, *J* = 1.2 Hz, 1H), 4.18–4.13 (m, 1H), 3.84–3.82 (m, 1H), 3.41 (s, 3H), 2.14 (d, *J* = 4.4 Hz, 1H), 1.69–1.63 (m, 2H), 1.48–1.28 (m, 8H), 0.88 (t, *J* = 7.2 Hz, 3H) ppm. ^13^C NMR (100 MHz, CDCl_3_): *δ* 138.0, 128.4, 128.2, 128.0, 109.3, 83.7, 81.0, 80.0, 76.7, 72.2, 55.8, 31.8, 30.2, 29.3, 26.2, 22.6, 14.1 ppm. IR (KBr) *ν*_max_ 3341, 3218, 3034, 2958, 2851, 1950, 1459, 1370, 1252, 1199, 1106, 1023, 960, 798, 747, 697, 605, 525, 467 C m^−1^. MR: 43 °C.

### *O*-((2*S*,3*R*,4*S*,5*R*)-4-(Benzyloxy)-5-hexyl-2-methoxytetrahydrofuran-3-yl)*S*-methyl carbonodithioate (3*α*)

To a stirred solution of 12α (6.0 g, 19.48 mmol, 1.0 eq.) in anhydrous THF (60 mL) under argon atmosphere was added 60% NaH (1.56 g, 38.96 mmol, 2.0 eq.) portion wise over a period of 15 minutes at 0 °C and stirred at same temperature for 1 h. Carbon disulphide (3.52 mL, 58.44 mmol, 3.0 eq.) was added and stirred the contents at same temperature for 1 h. Methyl iodide (3.63 mL, 58.44 mmol, 3.0 eq.) was added to above reaction mixture and stirred at 0 °C for 1 h. After completion of reaction, the reaction mixture was quenched with saturated aqueous NH_4_Cl solution (150 mL) and extracted with diethyl ether (200 mL). The organic layer was washed with water (100 mL), brine (100 mL), dried over anhydrous Na_2_SO_4_ and concentrated under reduced pressure to afford crude residue, which was purified by biotage chromatography using solvent gradient of 2.5% ethyl acetate in hexane to afford xanthate 3α (6.9 g, yield: 89%) as a pale yellow oil. *R*_f_ = 0.55 (10% EtOAc in hexane) [*α*]^25^_D_ = + 61.2° (*c* = 0.4% (w/v) in CHCl_3_). ^1^H NMR (400 MHz, CDCl_3_): *δ* 7.36–7.25 (m, 5H), 5.73 (t, *J* = 4.0 Hz, 1H), 5.23 (d, *J* = 4.4 Hz, 1H), 4.67 (d, *J* = 12.0 Hz, 1H), 4.51 (d, *J* = 12.0 Hz, 1H), 4.23–4.25 (m, 1H), 4.20–4.15 (m, 1H), 3.35 (s, 3H), 2.58 (s, 3H), 1.71–1.64 (m, 2H), 1.46–1.25 (m, 8H), 0.89 (t, *J* = 7.2 Hz, 3H) ppm. ^13^C NMR (100 MHz, CDCl_3_): *δ* 215.5, 137.6, 128.4, 127.8, 99.7, 86.3, 80.4, 76.7, 71.8, 55.6, 31.8, 29.3, 29.1, 25.9, 22.6, 19.4, 14.1 ppm. IR (KBr) *ν*_max_ 3328, 3032, 2959, 2918, 2857, 1876, 1735, 1594, 1502, 1457, 1369, 1268, 1207, 1134, 1079, 1033, 904, 746, 695, 605, 538, 441 C m^−1^. HRMS (ESI) calculated for C_22_H_34_0_4_S_2_ [M + H]^+^*m*/*z* = 399.1658, found: 399.1645.

### *O*-((2*R*,3*R*,4*S*,5*R*)-4-(Benzyloxy)-5-hexyl-2-methoxytetrahydrofuran-3-yl)*S*-methyl carbonodithioate (2β)

To a stirred solution of 12β (6.0 g, 19.48 mmol, 1.0 eq.) in anhydrous THF (60 mL) under argon atmosphere was added 60% NaH (1.56 g, 38.96 mmol, 2.0 eq.) portion wise over a period of 15 minutes at 0 °C and stirred the contents at same temperature for 1 h. Carbon disulphide (3.52 mL, 58.44 mmol, 3.0 eq.) was added and stirred at same temperature for 1 h. Methyl iodide (3.63 mL, 58.44 mmol, 3.0 eq.) was added to above reaction mixture and stirred at 0 °C for 1 h. After completion of reaction, the reaction mixture was quenched with saturated aqueous NH_4_Cl solution (150 mL) and extracted with diethyl ether (200 mL). The organic layer was washed with water (100 mL), brine (100 mL), dried over anhydrous Na_2_SO_4_ and concentrated under reduced pressure to afford crude residue, which was purified by biotage chromatography using solvent gradient of 2.5% ethyl acetate in hexane to afford xanthate 2β (7.1 g, yield: 92%) as a pale yellow oil. *R*_f_ = 0.5 (10% EtOAc in hexane) [*α*]^25^_D_ = −128.7° (*c* = 0.1% (w/v) in CHCl_3_). ^1^H NMR (400 MHz, CDCl_3_): *δ* 7.36–7.28 (m, 5H), 5.92 (s, 1H), 5.00 (s, 1H), 4.86 (d, *J* = 12.0 Hz, 1H), 4.63 (d, *J* = 12.4 Hz, 1H), 4.13–4.10 (m, 1H), 3.91 (d, *J* = 4.8 Hz, 1H), 3.44 (s, 3H), 2.58 (s, 3H), 1.77–1.67 (m, 2H), 1.34–1.21 (m, 8H), 0.88 (t, *J* = 6.8 Hz, 3H) ppm. ^13^C NMR (100 MHz, CDCl_3_): *δ* 214.6, 137.7, 128.3, 128.1, 127.8, 107.0, 88.1, 82.6, 79.8, 71.8, 55.8, 31.7, 29.7, 29.3, 26.1, 22.6, 19.3, 14.1 ppm. IR (KBr) *ν*_max_ 3746, 3680, 2920, 2856, 1790, 1741, 1649, 1560, 1507, 1459, 1344, 1270, 1209, 1069, 959, 805, 754, 697, 442 C m^−1^.

### (2*R*,3*R*,5*S*)-3-(Benzyloxy)-2-hexyl-52-methoxytetrahydrofuran (13)

#### Barton–McCombie deoxygenation

To a stirred solution of 3α (Xanthate) (6.0 g, 15.07 mmol, 1.0 eq.) in dry toluene (300 mL) under argon atmosphere was added tri butyl tin hydride (*n*-Bu_3_SnH, 7.31 mL, 27.13 mmol, 1.8 eq.) followed by azobis-isobutyronitrile (AIBN, 1.23 g, 7.54 mmol, 0.5 eq.) dissolved in toluene (60 mL) at 110 °C. The reaction mixture was stirred for 2 h at same temperature. After completion of reaction, the reaction mixture was concentrated under reduced pressure to afford crude residue, which was purified by biotage chromatography using solvent gradient of 3% ethyl acetate in hexane to afford deoxygenated product 13 (0.91 g, yield: 21%) as a colorless oil.*R*_f_ = 0.5 (10% EtOAc in hexane). [*α*]^25^_D_ = + 35.3° (*c* = 0.4% (w/v) in CHCl_3_). ^1^H NMR (400 MHz, CDCl_3_): *δ* 7.36–7.25 (m, 5H), 5.12–5.10 (m, 1H), 4.58 (d, *J* = 12.4 Hz, 1H), 4.39 (d, *J* = 12.4 Hz, 1H), 4.02–3.99 (m, 1H), 3.95–3.91 (m, 1H), 3.35 (s, 3H), 2.32–2.30 (m, 1H), 2.29–2.27 (m, 1H), 2.04–1.98 (m, 1H), 1.75 (q, 2H), 1.45–1.26 (m, 8H), 0.88 (t, *J* = 7.2 Hz, 3H) ppm. ^13^C NMR (100 MHz, CDCl_3_): *δ* 138.3, 128.3, 128.1, 127.7, 127.5, 127.4, 103.8, 80.5, 78.4, 76.7, 70.9, 55.1, 39.6, 31.8, 29.7, 29.4, 28.6, 26.3, 22.6, 14.1 ppm. IR(KBr) *ν*_max_ 3030, 2960, 2919, 2857, 1722, 1596, 1503, 1455, 1348, 1268, 1210, 1102, 1044, 990, 906, 808, 750, 696, 602, 437 C m^−1^. MS (EI): *m*/*z* (M + Na)^+^: 315.23.

### (2*R*,3*R*,5*R*)-3-(Benzyloxy)-2-hexyl-5-methoxytetrahydrofuran (16)

#### Barton–McCombie deoxygenation

To a stirred solution of 2β (Xanthate) (6.0 g, 15.07 mmol, 1.0 eq.) in dry toluene (300 mL) under argon atmosphere was added tri butyl tin hydride (*n*-Bu_3_SnH, 7.31 mL, 27.13 mmol, 1.8 eq.) followed by azobis-isobutyronitrile (AIBN, 1.23 g, 7.54 mmol, 0.5 eq.) dissolved in toluene (60 mL) at 110 °C. The reaction mixture was stirred for 2 h at same temperature. After completion of reaction, the reaction mixture was concentrated under reduced pressure to afford crude residue, which was purified by biotage chromatography using solvent gradient of 3% ethyl acetate in hexane to afford deoxygenated product 16 (3.93 g, yield: 89%) as a colorless oil.*R*_f_ = 0.4 (10% EtOAc in hexane). [*α*]^25^_D_ = −99.9° (*c* = 0.4% (w/v) in CHCl_3_). ^1^H NMR (400 MHz, CDCl_3_): *δ* 7.34–7.26 (m, 5H), 4.99–4.97 (m, 1H), 4.64 (d, *J* = 12.4 Hz, 1H), 4.45 (d, *J* = 12.0 Hz, 1H), 3.98–3.90 (m, 2H), 3.39 (s, 3H), 2.21–2.08 (m, 2H), 1.77–1.68 (m, 2H), 1.48–1.44 (m, 1H), 1.36–1.30 (m, 7H), 0.88 (t, *J* = 6.8 Hz, 3H) ppm. ^13^C NMR (100 MHz, CDCl_3_): *δ* 138.3, 128.3, 127.7, 127.5, 104.3, 82.2, 76.7, 71.3, 55.4, 37.9, 31.8, 30.0, 29.4, 26.3, 22.6, 14.1 ppm. IR(KBr) *ν*_max_ 3749, 3680, 3217, 3031, 2959, 2919, 2856, 1785, 1650, 1558, 1506, 1458, 1372, 1267, 1209, 1100, 1040, 946, 804, 751, 697, 605, 461 C m^−1^.

### (4*R*,5*R*)-4-(Benzyloxy)-5-hexyltetrahydrofuran-2-ol (14)

To a stirred solution of 13 (0.7 g, 2.4 mmol, 1.0 eq.) in 60% acetic acid: water (7 mL) was heated at 70 °C for 2 h. After completion of reaction, the reaction mixture was concentrated under reduced pressure to afford crude residue, which was purified by biotage chromatography using solvent gradient of 15% ethyl acetate in hexane to afford lactol 14 (0.52 g, yield: 79%) as a colorless oil. *R*_f_ = 0.2 (30% EtOAc in hexane) [*α*]^25^_D_ = −16.76° (*c* = 0.4% (w/v) in CHCl_3_). ^1^H NMR (400 MHz, CDCl_3_): *δ* 7.37–7.28 (m, 8H), 5.66 (bs, 1.0H), 5.37–5.33 (dd, *J* = 11.6 Hz, 4.8 Hz, 1H), 4.68–4.38 (m, 4.0H), 4.12–4.05 (m, 1H), 3.98 (t, *J* = 3.2 Hz, 1H), 3.88–3.84 (m, 1H), 3.63 (d, *J* = 11.6 Hz, 1H), 2.81 (bs, 1H), 2.37–2.28 (m, 2H), 2.05–1.96 (m, 2H), 1.84–1.63 (m, 5H), 1.44–1.29 (m, 9H), 0.90–0.87 (m, 6H) ppm. ^13^C NMR (100 MHz, CDCl_3_): *δ* 138.2, 137.4, 128.5, 128.3, 128.0, 127.8, 127.6, 127.5, 98.5, 97.4, 83.7, 80.9, 78.7, 78.2, 71.6, 71.0, 40.2, 39.1, 31.8, 30.5, 29.4, 28.7, 27.8, 26.8, 26.2, 22.6, 17.5, 14.1, 13.6 ppm. IR (KBr) *ν*_max_ 3411, 3030, 2961, 2920, 2857, 1722, 1503, 1455, 1349, 1266, 1203, 1103, 1027, 945, 900, 804, 747, 696, 603, 465, 421 C m^−1^. HRMS (ESI) calculated for C_19_H_30_0_3_ [M + Na]^+^*m*/*z*: 301.1774, found: 301.1772.

### (4*R*,5*R*)-4-(Benzyloxy)-5-hexyldihydrofuran-2(3*H*)-one (15)

To a stirred solution of 14 (0.50 g, 1.80 mmol, 1.0 eq.) in DCM (5 mL) under argon atmosphere was added diacetoxy iodo benzene (BAIB, 0.69 g, 2.16 mmol, 1.2 eq.), followed by (2,2,6,6-tetramethylpiperidin-1-yl)oxyl (TEMPO, 0.06 g, 0.36 mmol, 0.2 eq.) at 0 °C and stirred the contents at room temperature for 1 h. After completion of reaction, the reaction mixture was diluted with DCM (20 mL), washed with saturated sodium bicarbonate solution (20 mL), water (20 mL), brine (20 mL), dried over anhydrous Na_2_SO_4_ and concentrated under reduced pressure to afford crude residue, which was purified by biotage chromatography using solvent gradient of 7% ethyl acetate in hexane to afford lactone 15 (0.41 g, yield: 84%) as a colorless oil. *R*_f_ = 0.5 (20% EtOAC in hexane). [*α*]^25^_D_ = −8.3° (*c* = 0.4% (w/v) in CHCl_3_). ^1^H NMR (400 MHz, CDCl_3_): *δ* 7.38–7.30 (m, 5H), 4.62 (d, 12.0 Hz, 3H), 4.44–4.39 (m, 2H), 4.18–4.15 (m, 1H), 2.72–2.60 (m, 1H), 1.92–1.82 (m, 1H), 1.81–1.73 (m, 1H), 1.48–1.30 (m, 8H), 0.89 (t, *J* = 6.8 Hz, 3H) ppm. ^13^C NMR (100 MHz, CDCl_3_): *δ* 175.2, 137.2, 128.5, 128.0, 127.6, 84.3, 77.3, 75.0, 71.3, 35.8, 31.6, 29.1, 28.5, 25.4, 22.5, 14.0 ppm. IR (KBr) *ν*_max_ 3746, 3680, 2921, 2857, 1833, 1778, 1649, 1561, 1505, 1459, 1401, 1346, 1267, 1205, 1154, 1092, 1030, 970, 912, 753, 696, 602, 551, 446 C m^−1^. HRMS (ESI) calculated for C_19_H_28_0_3_ [M + H]^+^*m*/*z* = 277.1798, found: 277.1795.

### (4*R*,5*R*)-5-Hexyl-4-hydroxydihydrofuran-2(3*H*)-one 1 (Japanese orange fly lactone)

To a stirred solution of 15 (0.2 g, 0.72 mmol, 1.0 eq.) in Methanol (3 mL) under argon atmosphere was added 10% Pd/C (50 mg) and the reaction mixture was stirred at room temperature for 16 h in an atmosphere of hydrogen created by evacuative displacement of air by hydrogen (balloon). After completion of reaction, the reaction mixture was filtered through Celite bed and the filtrate was concentrated under reduced pressure to afford crude residue, which was purified by biotage chromatography using solvent gradient of 17% ethyl acetate in hexane to afford Japanese orange fly lactone (1) (0.11 g, 82%) as a colorless solid. *R*_f_ = 0.1 (30% EtOAc in hexane) [*α*]^25^_D_ = + 52.0° (*c* = 0.76% (w/v) in CHCl_3_). ^1^H NMR (400 MHz, CDCl_3_): *δ* 4.47 (t, *J* = 4.8 Hz, 1H), 4.39–4.35 (m, 1H), 2.81 (dd, 17.6 Hz, 5.6 Hz, 1H), 2.57 (dd, 17.6 Hz, 0.8 Hz, 1H), 2.35 (brs, 1H), 1.90–1.82 (m, 1H), 1.77–1.68 (m, 1H), 1.54–1.38 (m, 8H), 0.88 (t, *J* = 7.2 Hz, 3H). ^13^C NMR (100 MHz, CDCl_3_): *δ* 176.1, 85.1, 68.9, 39.5, 31.6, 29.1, 28.2, 25.5, 22.5, 14.0 ppm. IR (KBr) *ν*_max_ 3746, 3432, 2964, 2922, 2859, 1764, 1647, 1509, 1462, 1403, 1348, 1270, 1208, 1165, 1083, 1021, 963, 908, 756, 677, 560 C m^−1^; HRMS (ESI) calculated for C_12_H_22_0_3_ [M + H]^+^*m*/*z* = 187.1329, found: 187.1326.

## Conclusions

In summary, we have synthesized Japanese orange fly lactone in a concise and efficient manner using Barton–McCombie deoxygenation, Wittig olefination and TEMPO mediated lactonization as key steps. This chiral pool approach reveals the behavior of the furanose anomeric isomers towards the Barton– McCombie reaction.

## Conflicts of interest

There are no conflicts to declare.

## Supplementary Material

RA-012-D2RA04531A-s001
