# Supplementary material for: Study of the β-oxygen effect in the Barton–McCombie reaction for the total synthesis of (4R,5R)-4-hydroxy-γ-decalactone (Japanese orange fly lactone): a carbohydrate based approach
Source: RSC Adv. 2022 Sep 7;12(39):25520–7. doi: 10.1039/d2ra04531a (PMC9449999; doi:10.1039/d2ra04531a)
Supplement: RA-012-D2RA04531A-s001 [file RA-012-D2RA04531A-s001.pdf]

Electronic Supplementary Material (ESI) for RSC Advances.
This journal is © The Royal Society of Chemistry 2022

Supporting Information for

Study of β-Oxygen effect in the Barton–McCombie Reaction for the Total synthesis of (4*R*,5*R*)-4-hydroxy-γ-decalactone (Japanese orange fly lactone): A Carbohydrate based Approach

Janardana Reddi Desireddi, * [a,b] Mallikarjuna Rao Mora, [c] Kiran Kumar Murahari, [a] Rajasekhar Reddy Nimma Reddy, [a] Thirupathi Mothe , [b] Arun Kumar Lingala, [b] Bhimcharan Maiti , [a] and Ravinder Manchal, * [b]

[a] Aragen life sciences Private Limited (formerly known as GVK Biosciences Private Limited) Medicinal Chemistry Division, 28A, IDA Nacharam, Hyderabad - 500076, Telangana, India.

[b] Department of Chemistry Chaitanya (Deemed to be University) Warangal-506001, Telangana, India.

[c] Accrete Pharmaceuticals Private Limited, Tangadpalli Village, Choutuppal Mandal,Yadadri Bhuvanagiri District-508252, Telangana, India

Table of Contents

1. Comparative study table of Japanese Orange fly Lactone 2

2. Copies of ^{1}H, ^{13}CNMR and FT-IR, spectra's 3-26

Table1: NMR chemical shift values of Japanese orange fly lactone in comparison to the isolated compound.

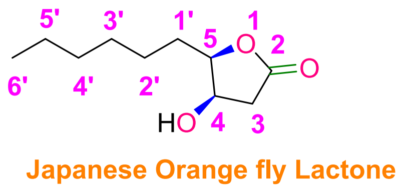

1

Position	Natural Japanese Orange Fly Lactone 1HNMR data in ppm (multiplicity, J in Hz)	Synthetic Japanese Orange Fly Lactone 1HNMR data in ppm (multiplicity, J in Hz) Hz)	Natural Japanese Orange Fly Lactone ^{13}CNMR data in ppm (multiplicity, J in Hz)	Synthetic Japanese Orange Fly Lactone ^{13}CNMR data in ppm (multiplicity, J in Hz) Hz)
2			175.3	176.1
3	2.80 (dd, 17.6, 5.6)<br>2.56 (dd, 17.6, 1.0)	2.81 (dd, 17.6, 5.6)<br>2.56 (dd, 17.6, 0.8)	39.6	39.5
4	4.49 (m)	4.47 (m)	69.3	68.9
5	4.37 (m)	4.39, (m)	84.7	85.1
1’	1.88 (m)	1.89-1.82 (m)	28.4	28.2
2’	1.71 (m)	1.77-1.68 (m)	25.7	25.5
3’	1.55-1.25 (m)	1.54-1.25 (m)	29.3	29.1
4’	1.55-1.25 (m)	1.54-1.25 (m)	22.7	22.5
5’	1.55-1.25 (m)	1.54-1.25 (m)	31.8	31.6
6’	0.90, t (7.0)	0.88, t (7.2)	14.2	14.0

Spectral data:

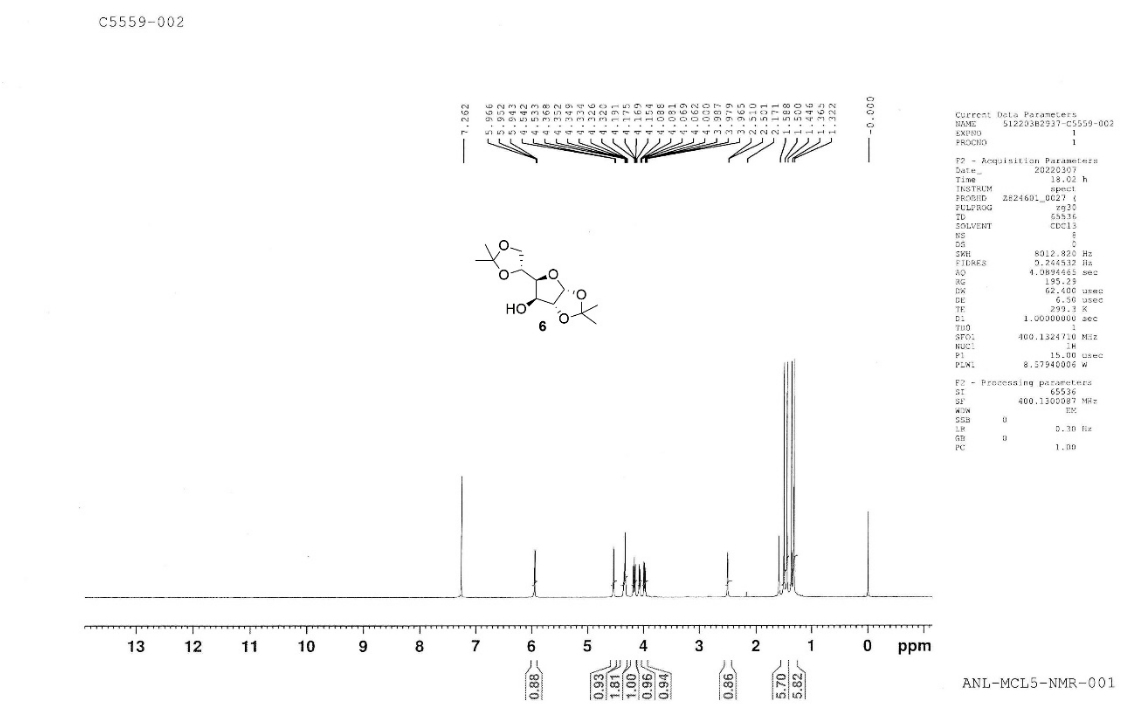

C5559-018-bn

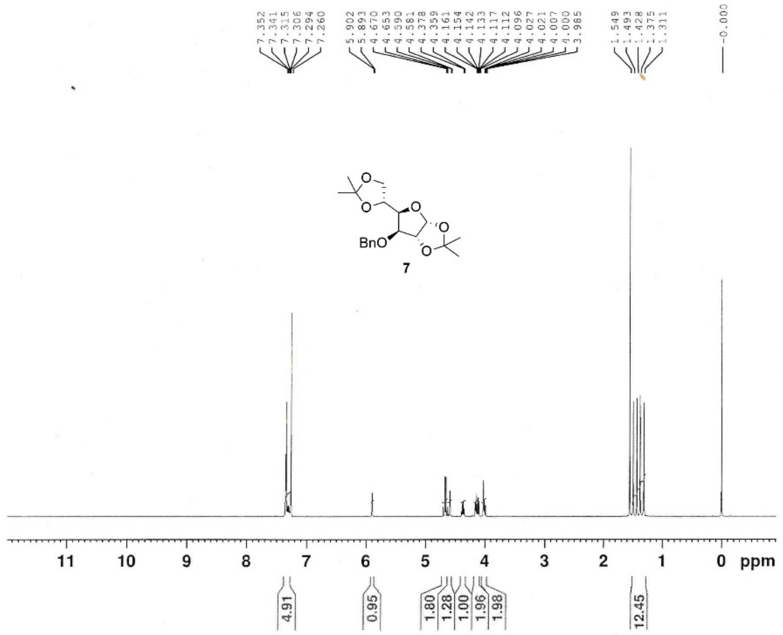


```
Current Data Parameters
NAME      512110B7147-C5559-018-bn
EXPNO              1
PROCNO             1

F2 - Acquisition Parameters
Date_           20211008
Time              17.47 h
INSTRUM  Avance Neo Nanobay 400
PROBHD   Z163739_0439 (
PULPROG             zg30
TD                 65536
SOLVENT            CDC13
NS                     8
DS                     0
SWH             8196.722 Hz
FIDRES          0.250144 Hz
AQ             3.9976959 sec
RG                   101
DW                61.000 usec
DE                 13.89 usec
TE                 298.2 K
D1            1.00000000 sec
TD0                    1
SFO1         400.2324714 MHz
NUC1                  1H
P0                  2.67 usec
P1                  8.00 usec
PLW1         22.80999947 W

F2 - Processing parameters
SI                 65536
SF           400.2300103 MHz
WDW                   EM
SSB                    0
LB                  0.30 Hz
GB                     0
PC                  1.00
```

ANL-MCL5-NMR-007

aragen

Aragen Life Sciences Private Limited

Analytical - Discovery Chemistry

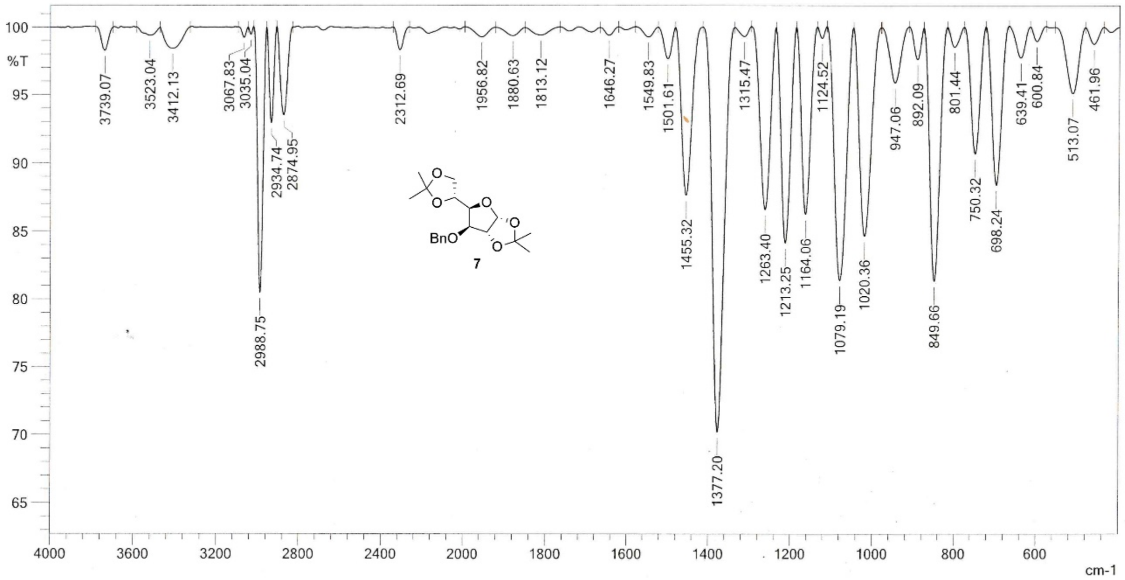

Sample Code : C5559-018-obn

Date : 21-Sep-2021 12:52:39 PM

Sample ID : 512109D8826

Instrument ID : ANL-MCL1-FT-IR-002

C5559-019-dp

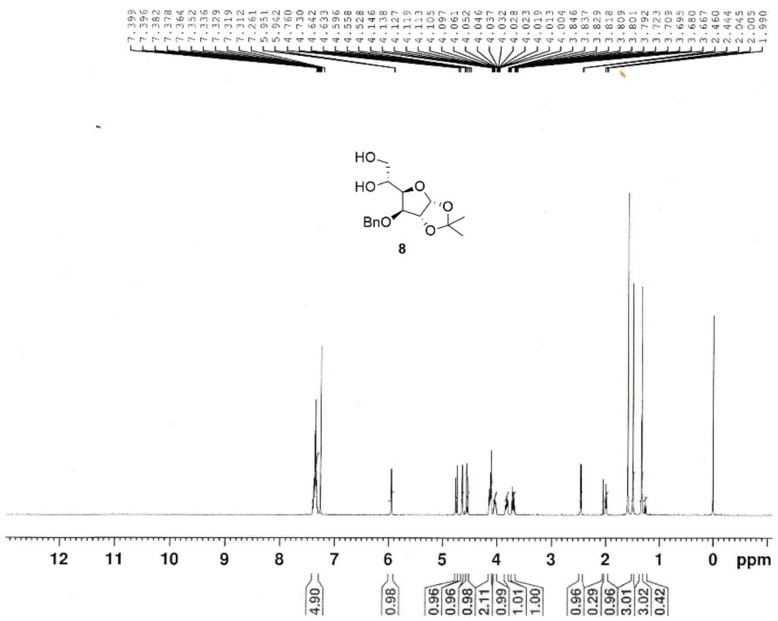

Current Data Parameters
NAME 512110B7148-C5559-019-dp
EXPNO 1
PROCNO 1

F2 - Acquisition Parameters
Date_ 20211008
Time 17.43 h
INSTRUM Avance Neo Nanobay 400
PROBHD Z163739_0439 (
PULPROG zg30
TD 65536
SOLVENT CDCl3
NS 8
DS 0
SWH 8196.722 Hz
FIDRES 0.250144 Hz
AQ 3.9976959 sec
RG 101
DW 61.000 usec
DE 13.89 usec
TE 298.1 K
D1 1.00000000 sec
TD0 1
SFO1 400.2324714 MHz
NUC1 1H
P0 2.67 usec
P1 8.00 usec
PLW1 22.80999947 W

F2 - Processing parameters
SI 65536
SF 400.2300100 MHz
WDW EM
SSB 0
LB 0.30 Hz
GB 0
PC 1.00

ANL-MCL5-NMR-007

aragen

Aragen Life Sciences Private Limited
Analytical - Discovery Chemistry

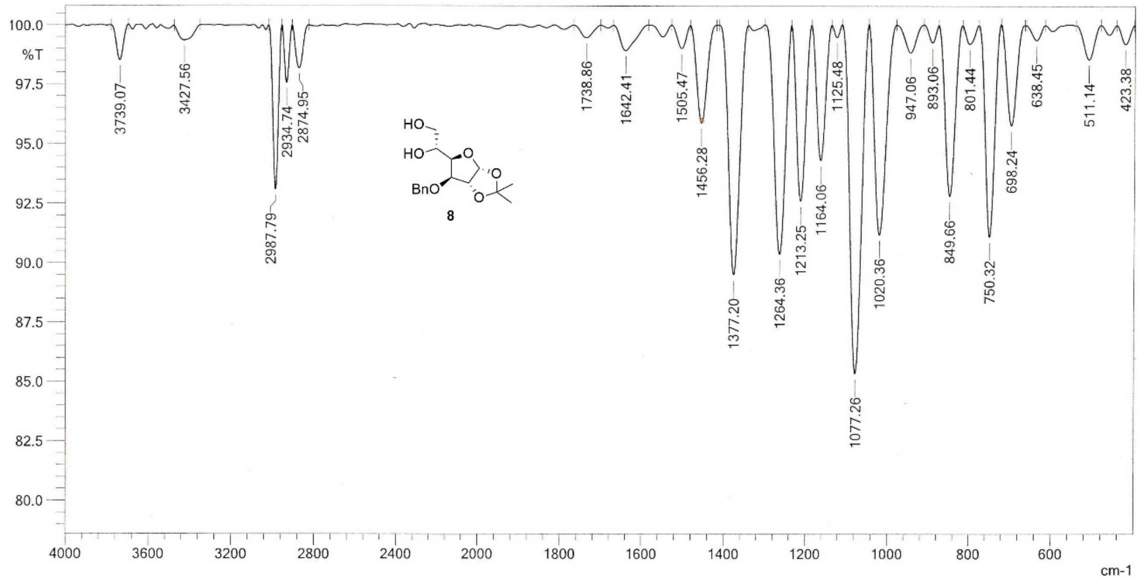

Sample Code : C5559-019-dp

Date : 21-Sep-2021 12:57:06 PM

Sample ID : 512109D8827

Instrument ID : ANL-MCL1-FT-IR-002

aragen

Aragen Life Sciences Private Limited

Analytical - Discovery Chemistry

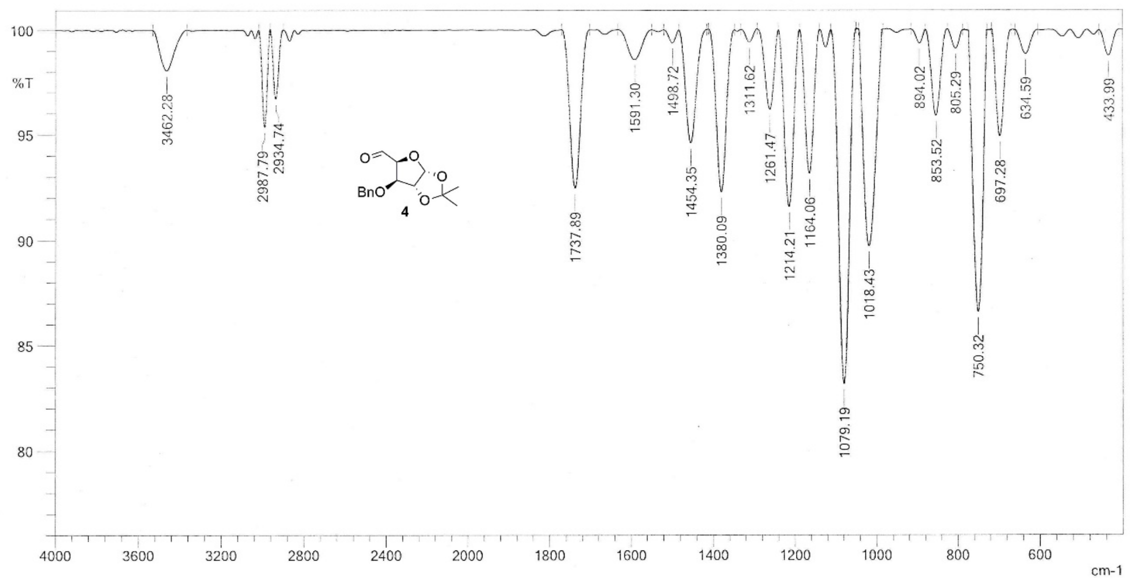

Sample Name : C5559-020-ald

Sample ID : 512205E9461

Date : 24-May-2022 12:23:09 PM

Instrument ID : ANL-MCL1-FT-IR-002

C5559-021-wi

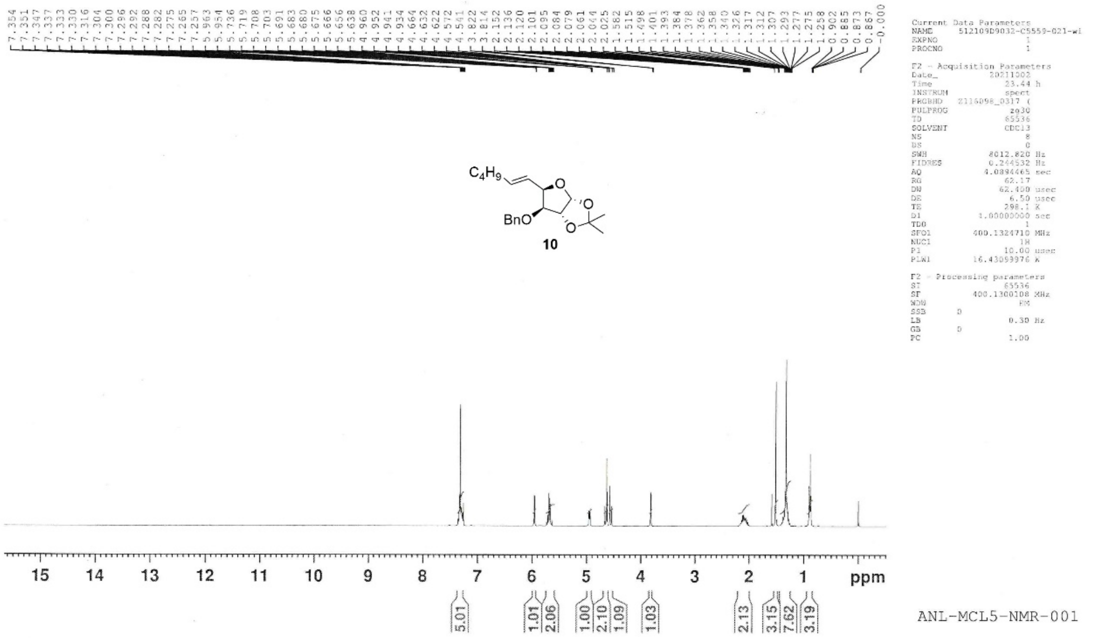

ANL-MCL5-NMR-001

C5559-021-wi

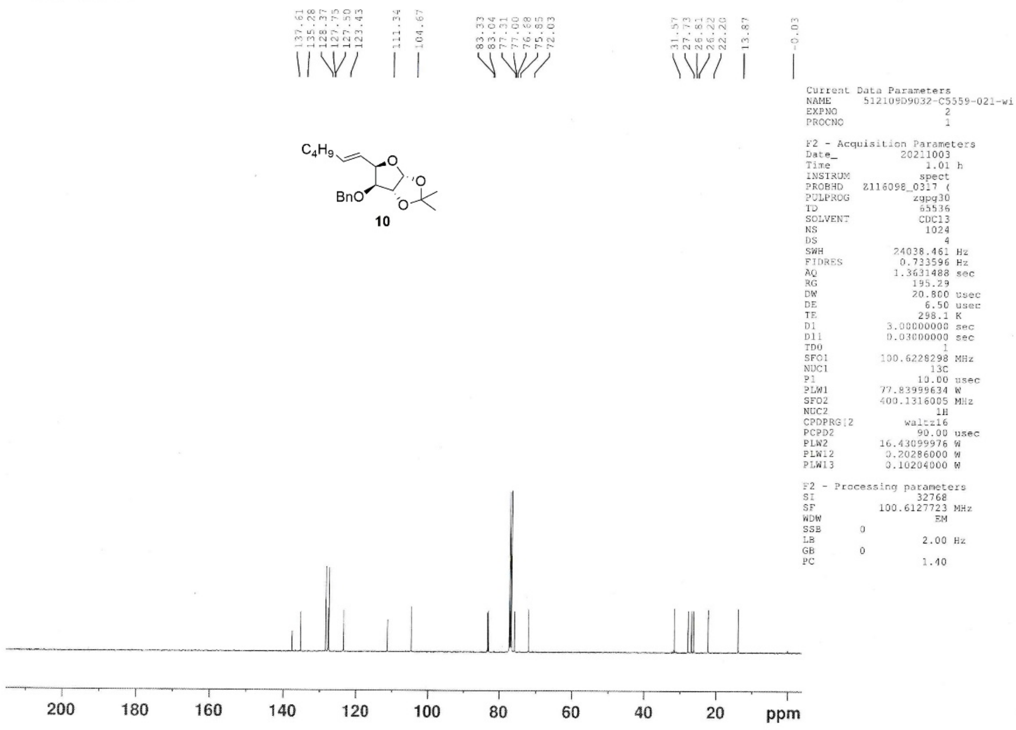


```
Current Data Parameters
NAME      512109D9032-C5559-021-wi
EXPNO                    2
PROCNO                   1

F2 - Acquisition Parameters
Date_             20211003
Time                  1.01 h
INSTRUM              spect
PROBHD   Z116098_0317 (
PULPROG             zgpg30
TD                   65536
SOLVENT              CDC13
NS                    1024
DS                       4
SWH              24038.461 Hz
FIDRES            0.733596 Hz
AQ               1.3631488 sec
RG                  195.29
DW                  20.800 usec
DE                    6.50 usec
TE                   298.1 K
D1              3.00000000 sec
D11             0.03000000 sec
TD0                      1
SFO1           100.6228298 MHz
NUC1                   13C
P1                   10.00 usec
PLW1           77.83999634 W
SFO2           400.1316005 MHz
NUC2                    1H
CPDPRG[2           waltz16
PCPD2                90.00 usec
PLW2           16.43099976 W
PLW12           0.20286000 W
PLW13           0.10204000 W

F2 - Processing parameters
SI                   32768
SF             100.6127723 MHz
WDW                     EM
SSB      0
LB                    2.00 Hz
GB       0
PC                    1.40
```

aragen

Aragen Life Sciences Private Limited
Analytical - Discovery Chemistry

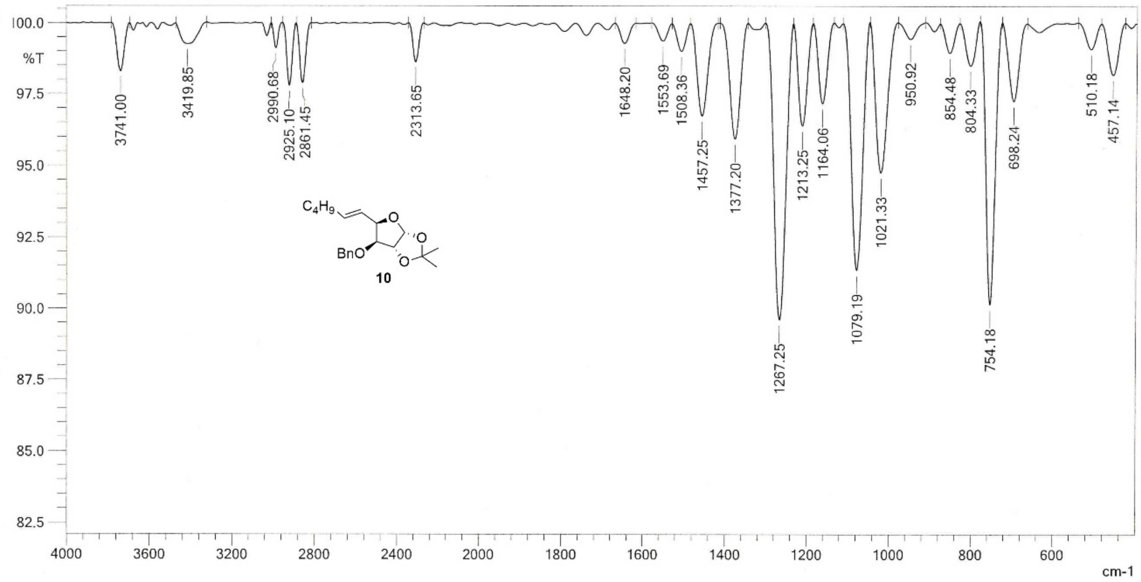

Sample Code : C5559-021-wi

Date : 21-Sep-2021 02:21:11 PM

Sample ID : 512109D8828

Instrument ID : ANL-MCL1-FT-IR-002

C5559-022-pdc

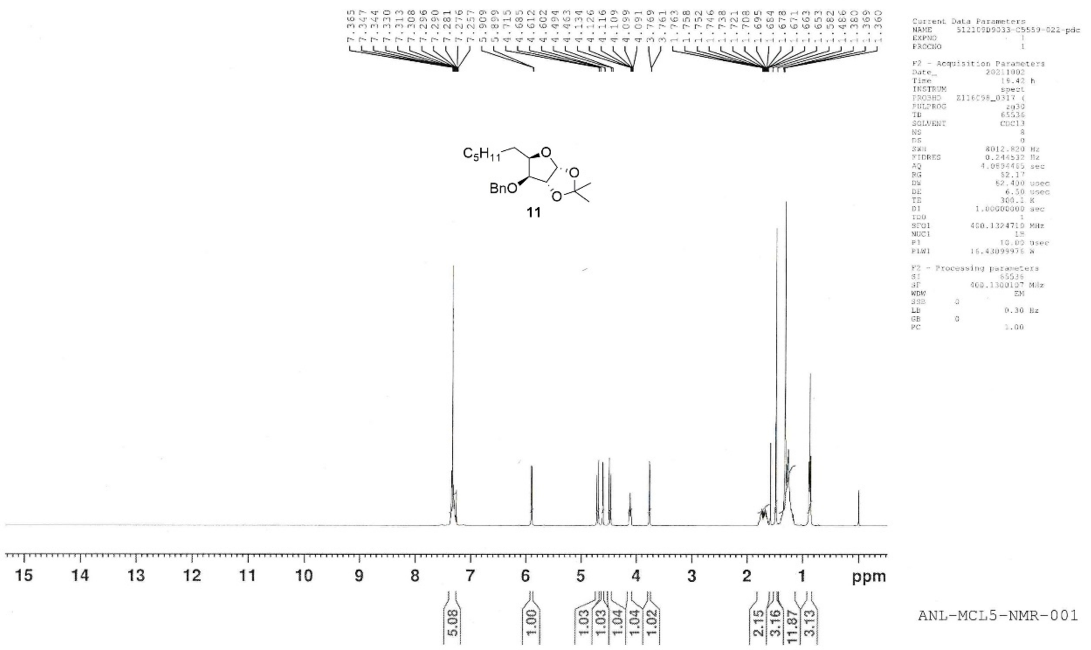

ANL-MCL5-NMR-001

C5559-022-pdc

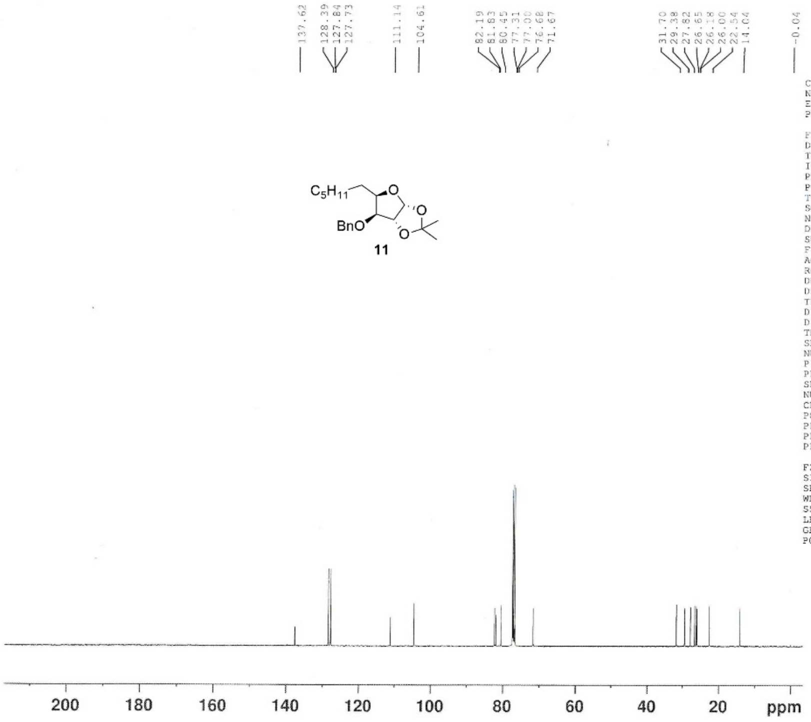

11

Current Data Parameters
NAME 512109D9033-C5559-022-pdc
EXPNO 2
PROCNO 1

F2 - Acquisition Parameters
Date_ 20211002
Time 20.59 h
INSTRUM spect
PROBHD Z116098_0317 (
PULPROG zgpg30
TD 65536
SOLVENT CDCl3
NS 1024
DS 4
SWH 24038.461 Hz
FIDRES 0.733596 Hz
AQ 1.3631488 sec
RG 195.29
DW 20.800 usec
DE 6.50 usec
TE 298.3 K
D1 3.00000000 sec
D11 0.03000000 sec
TD0 1
SFO1 100.6228298 MHz
NUC1 13C
P1 10.00 usec
PLW1 77.83999634 W
SFO2 400.1316005 MHz
NUC2 1H
CPDPRG[2 waltz16
PCPD2 90.00 usec
PLW2 16.43099976 W
PLW12 0.20286000 W
PLW13 0.10204000 W

F2 - Processing parameters
SI 32768
SF 100.6127718 MHz
WDW EM
SSB 0
LB 2.00 Hz
GB 0
PC 1.40

aragen

Aragen Life Sciences Private Limited
Analytical - Discovery Chemistry

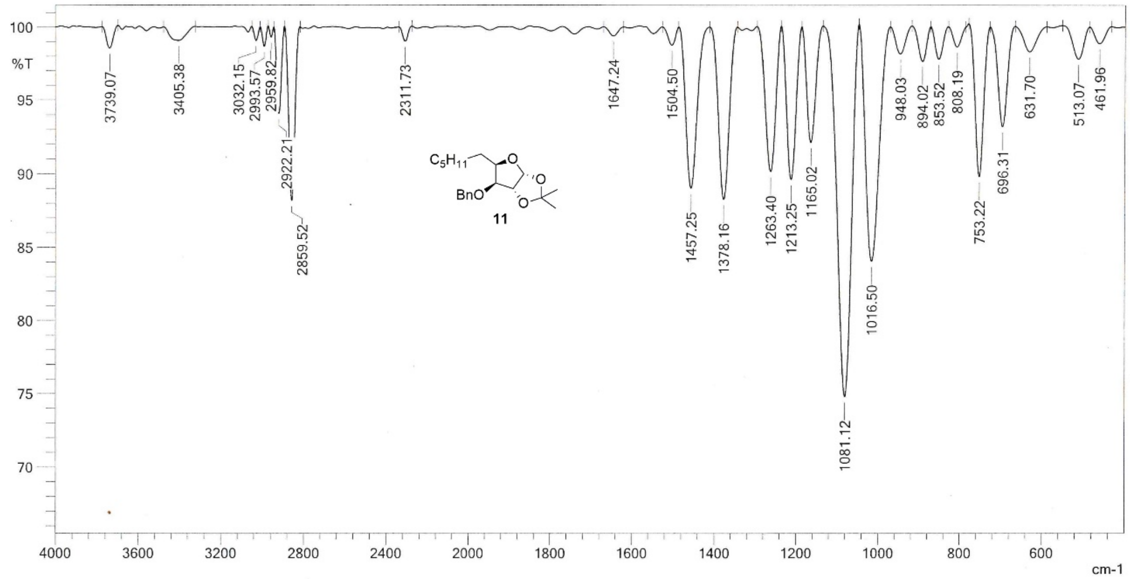

Sample Code : C5559-022-pdc

Date : 21-Sep-2021 02:28:21 PM

Sample ID : 512109D8829

Instrument ID : ANL-MCL1-FT-IR-002

C5559-023-AMB-1

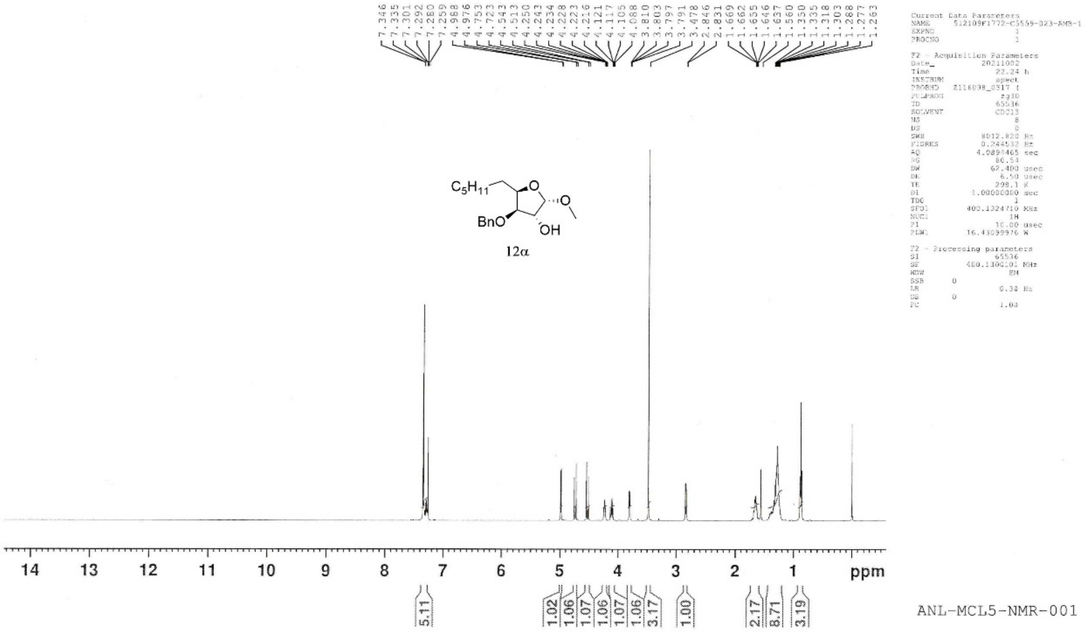

ANL-MCL5-NMR-001

023-AMB-1 NOESY1D
Selective band center: 4.99 (ppm); width: 24.6 (Hz)

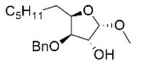

12α

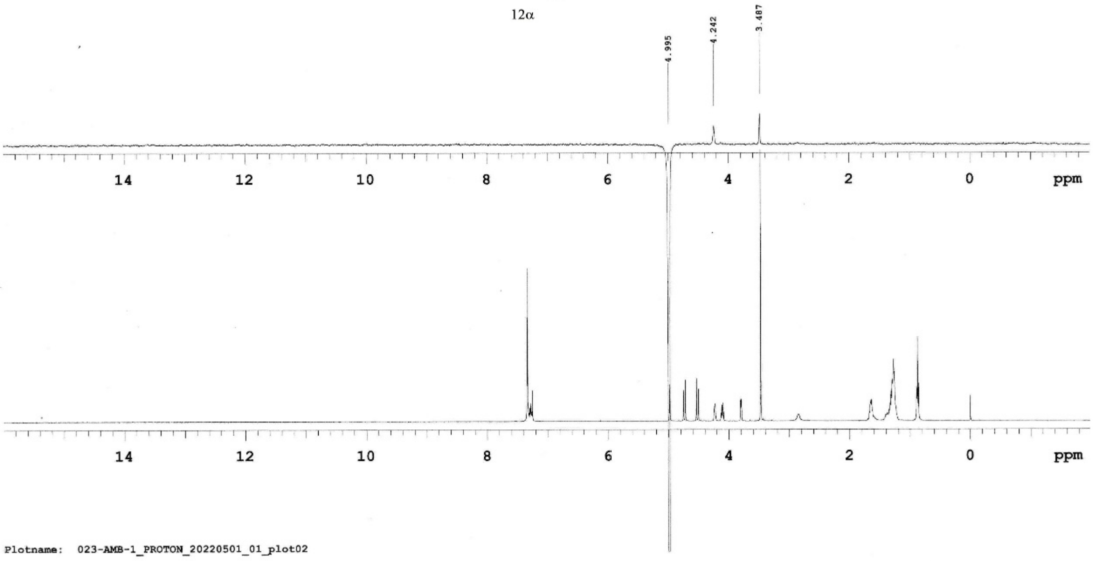

Plotname: 023-AMB-1_PROTON_20220501_01_plot02

C5559-023-AMB-1

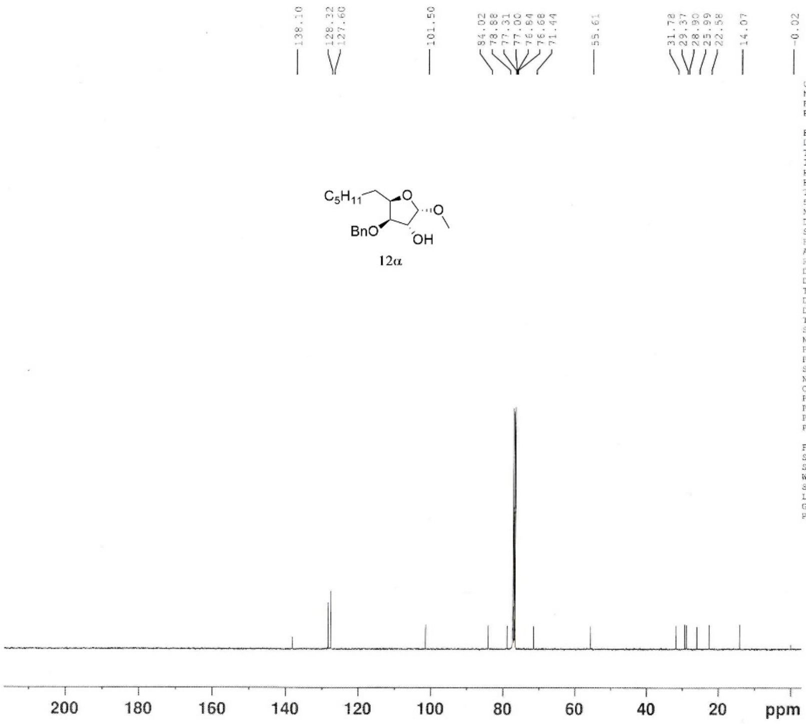

12α

Current Data Parameters
NAME 512109F1772-C5559-023-AMB-1
EXPNO 2
PROCNO 1

F2 - Acquisition Parameters
Date_ 20211002
Time 23.41 h
INSTRUM spect
PROBHD Z116098_0317 (
PULPROG zgpg30
TD 65536
SOLVENT CDCl3
NS 1024
DS 4
SWH 24038.461 Hz
FIDRES 0.733596 Hz
AQ 1.3631488 sec
RG 195.29
DW 20.800 usec
DE 6.50 usec
TE 298.1 K
D1 3.00000000 sec
D11 0.03000000 sec
TD0 1
SFO1 100.6228298 MHz
NUC1 13C
P1 10.00 usec
PLW1 77.83999634 W
SFO2 400.1316005 MHz
NUC2 1H
CPDPRG[2 waltz16
PCPD2 90.00 usec
PLW2 16.43099976 W
PLW12 0.20286000 W
PLW13 0.10204000 W

F2 - Processing parameters
SI 32768
SF 100.6127707 MHz
WDW EM
SSB 0
LB 2.00 Hz
GB 0
PC 1.40

Aragen Life Sciences Private Limited
Analytical - Discovery Chemistry

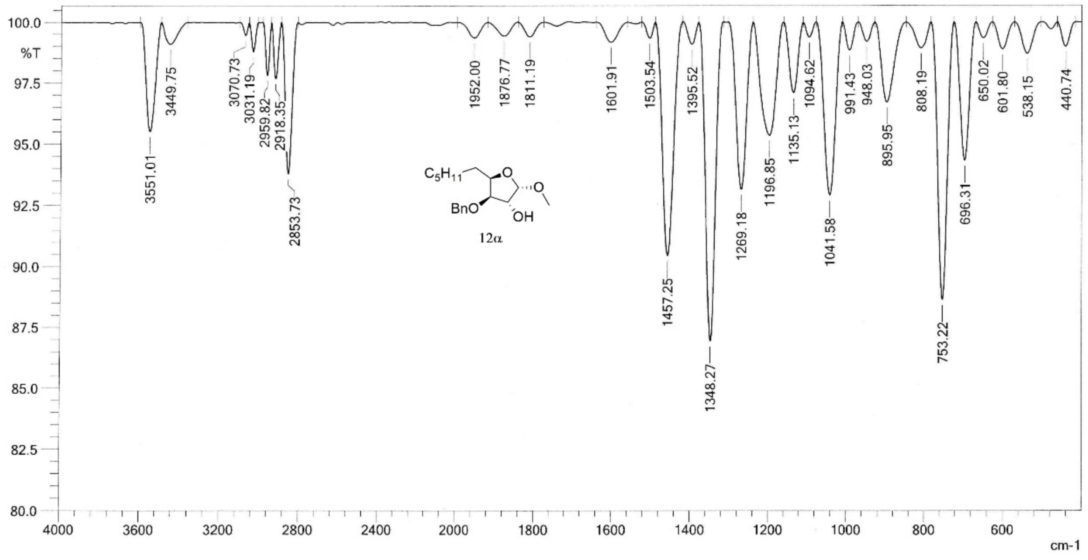

Sample Name : C5559-023-AMB-1

Sample ID : 512204A0604

Date : 01-Apr-2022 05:36:27 PM

Instrument ID : ANL-MCL1-FT-IR-002

C5559-023-AMB-2

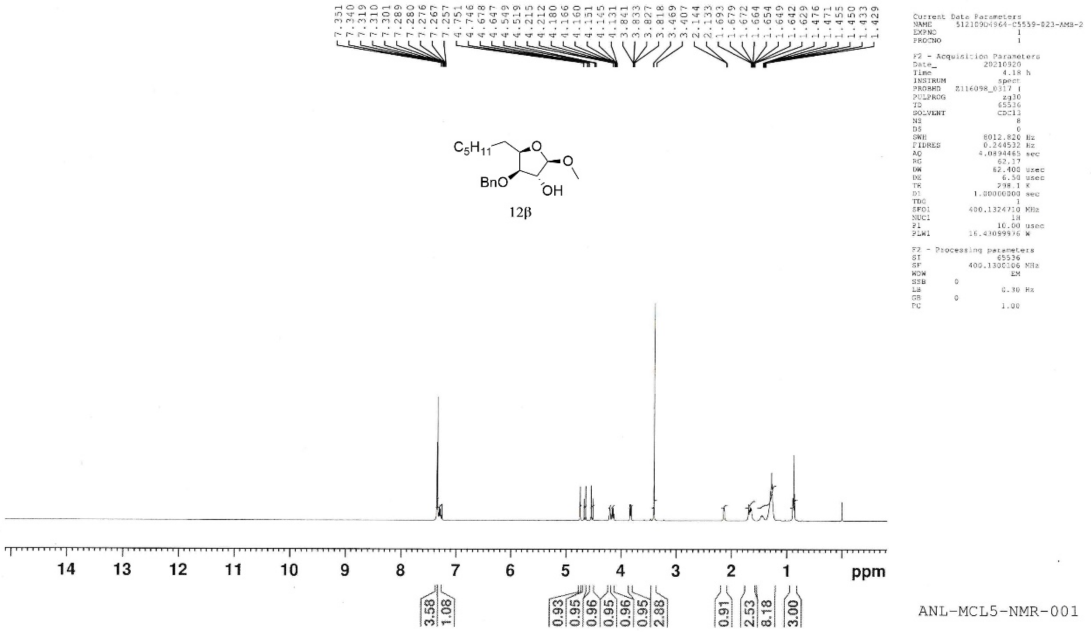

ANL-MCL5-NMR-001

023-AMB-2 NOESY1D
Selective band center: 4.74 (ppm); width: 18.8 (Hz)

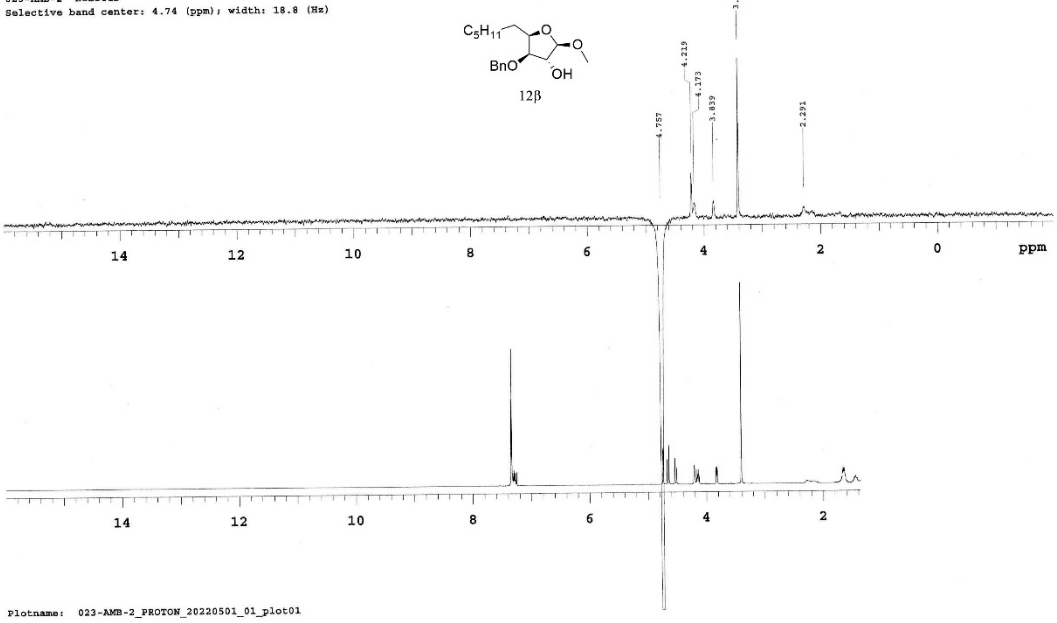

Plotname: 023-AMB-2_PROTON_20220501_01_plot01

C5559-023-AMB-2

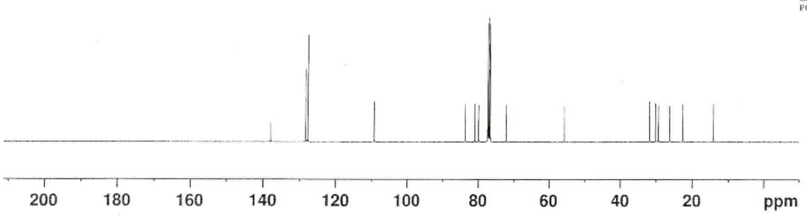

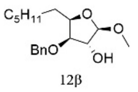

12β

Current Data Parameters
NAME 512109D4964-C5559-023-AMB-2
EXPNO 2
PROCNO 1

F2 - Acquisition Parameters
Date_ 20210920
Time 6.47 h
INSTRUM spect
PROBHD Z116098_0317 (
PULPROG zgpg30
TD 65536
SOLVENT CDCl3
NS 2000
DS 4
SWH 24038.461 Hz
FIDRES 0.733596 Hz
AQ 1.3631488 sec
RG 195.29
DW 20.800 usec
DE 6.50 usec
TE 298.2 K
D1 3.00000000 sec
D11 0.03000000 sec
TD0 1
SFO1 100.6228298 MHz
NUC1 13C
P1 10.00 usec
PLW1 77.83999634 W
SFO2 400.1316005 MHz
NUC2 1H
CPDPRG[2 waltz16
PCPD2 90.00 usec
PLW2 16.43099976 W
PLW12 0.20286000 W
PLW13 0.10204000 W

F2 - Processing parameters
SI 32768
SF 100.6127726 MHz
WDW EM
SSB 0
LB 2.00 Hz
GB 0
PC 1.40

Aragen Life Sciences Private Limited
Analytical - Discovery Chemistry

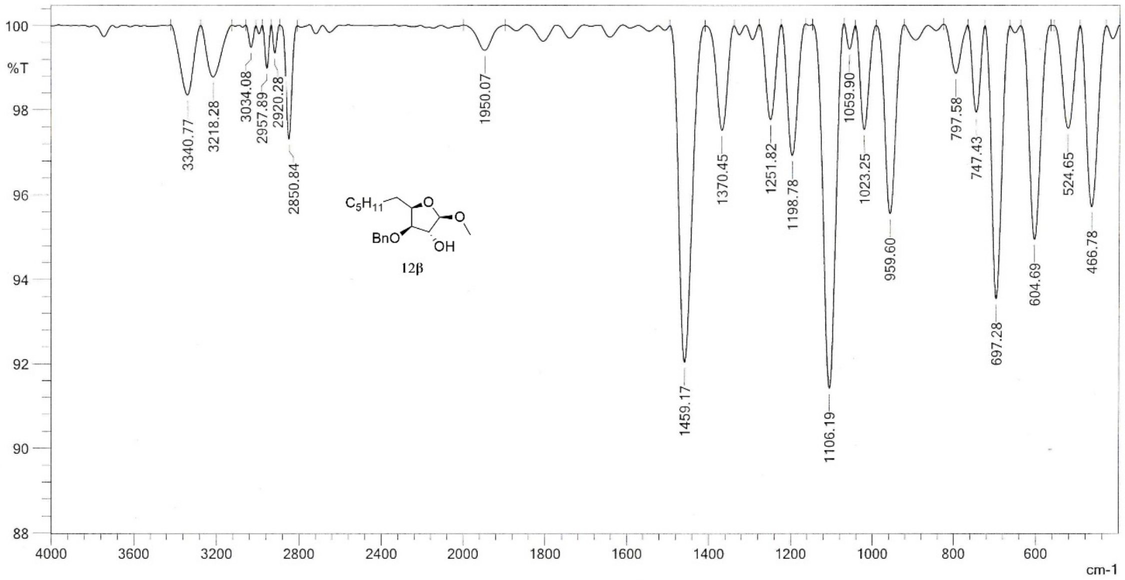

Sample Code : C5559-023-amb-2

Date : 21-Sep-2021 02:45:57 PM

Sample ID : 512109D8830

Instrument ID : ANL-MCL1-FT-IR-002

C5559-024-XN-1

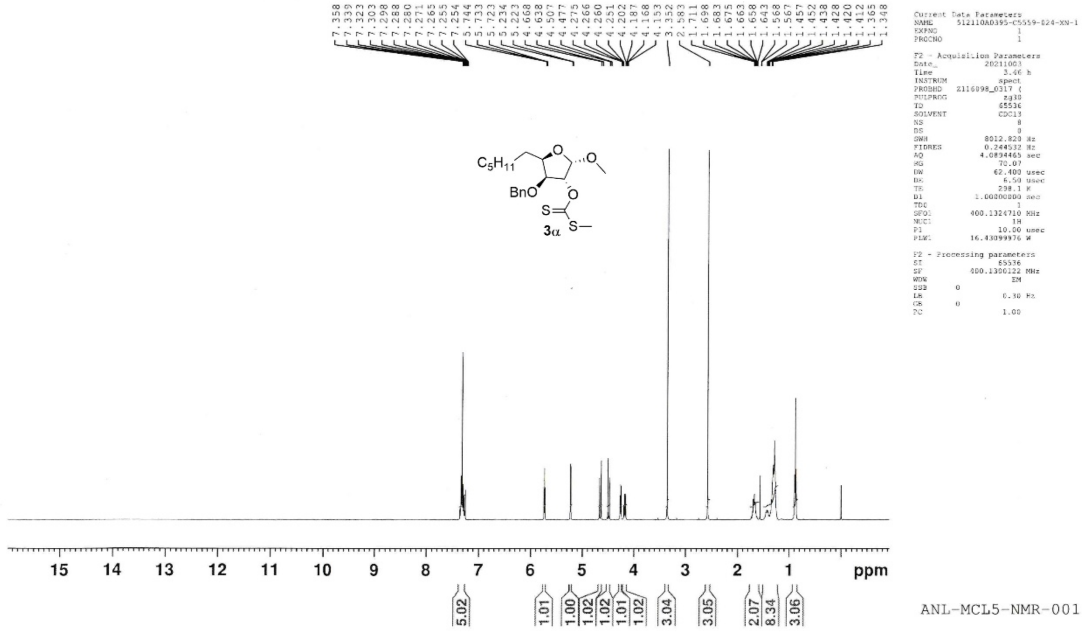

ANL-MCL5-NMR-001

C5559-024-XN-1

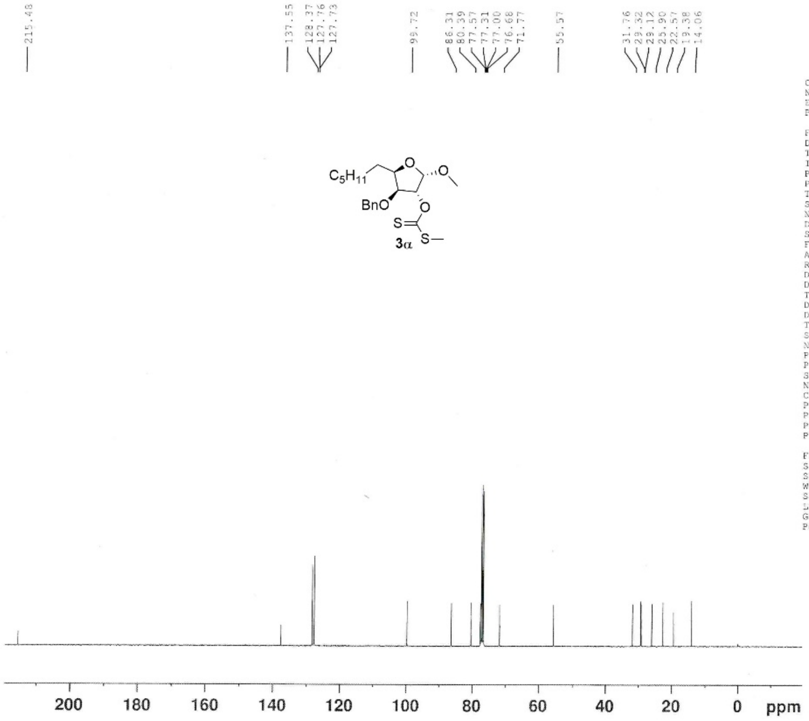


```
Current Data Parameters
NAME     512110A0395-C5559-024-XN-1
EXPNO                 2
PROCNO                1

F2 - Acquisition Parameters
Date_          20211003
Time               5.03 h
INSTRUM           spect
PROBHD   Z116098_0317 (
PULPROG          zgpg30
TD                65536
SOLVENT           CDCl3
NS                 1024
DS                    4
SWH           24038.461 Hz
FIDRES         0.733596 Hz
AQ            1.3631488 sec
RG               195.29
DW               20.800 usec
DE                 6.50 usec
TE                298.2 K
D1           3.00000000 sec
D11          0.03000000 sec
TD0                   1
SFO1        100.6228298 MHz
NUC1                13C
P1                10.00 usec
PLW1        77.83999634 W
SFO2        400.1316005 MHz
NUC2                 1H
CPDPRG[2        waltz16
PCPD2             90.00 usec
PLW2        16.43099976 W
PLW12        0.20286000 W
PLW13        0.10204000 W

F2 - Processing parameters
SI                32768
SF          100.6127725 MHz
WDW                  EM
SSB      0
LB                 2.00 Hz
GB       0
PC                 1.40
```

aragen

Aragen Life Sciences Private Limited
Analytical - Discovery Chemistry

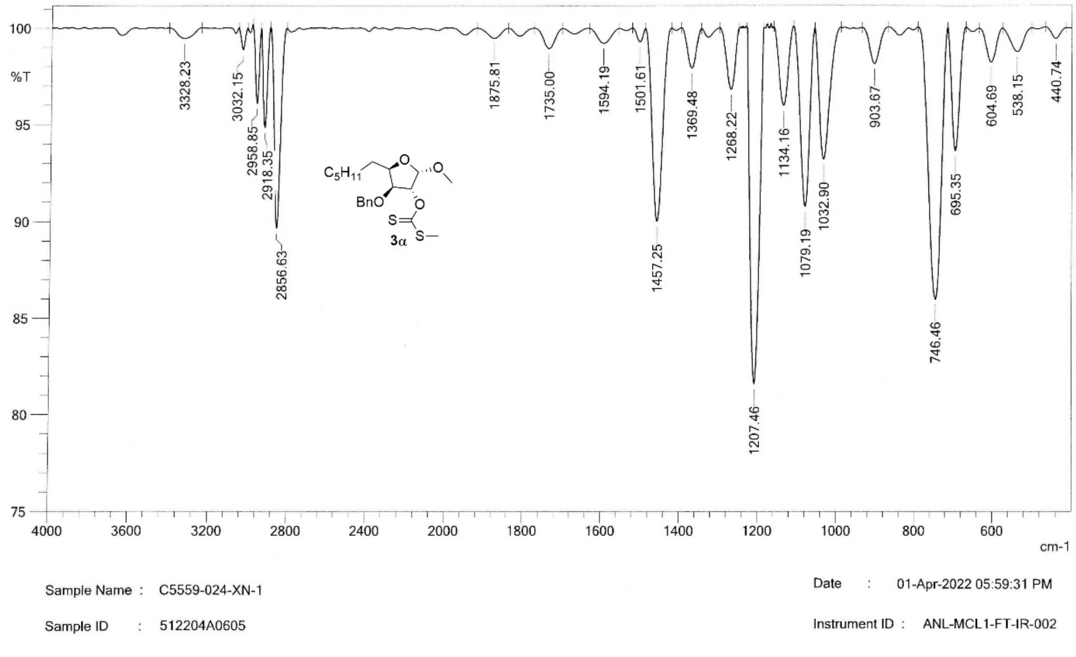

Sample Name : C5559-024-XN-1

Sample ID : 512204A0605

Date : 01-Apr-2022 05:59:31 PM

Instrument ID : ANL-MCL1-FT-IR-002

C5559-024-xn-2

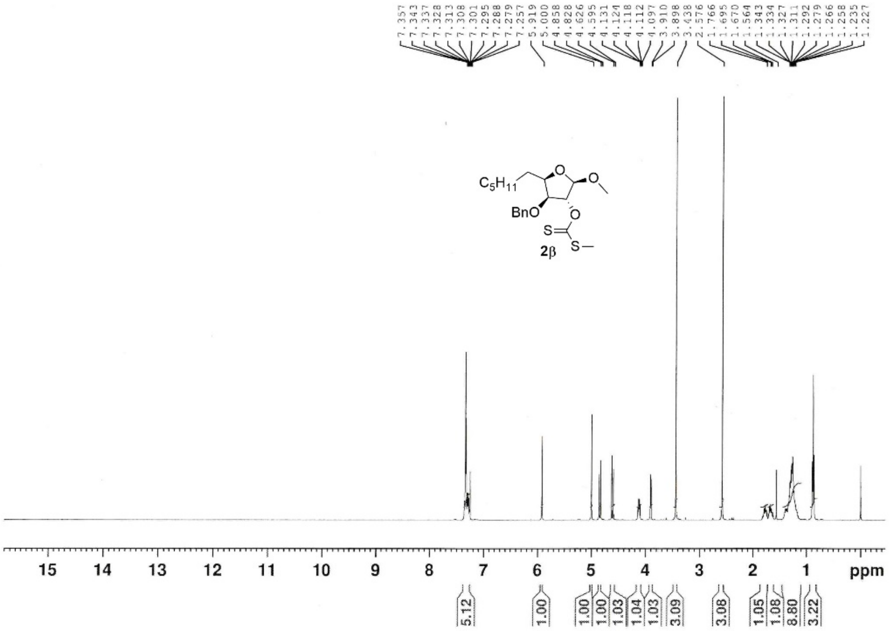

Current Data Parameters
NAME 512109D9034-C5559-024-xn-2
EXPNO 1
PROCNO 1

F2 - Acquisition Parameters
Date_ 20211003
Time 1.05 h
INSTRUM spect
PROBHD Z116098_0317 (
PULPROG zg30
TD 65536
SOLVENT CDCl3
NS 8
DS 0
SWH 8012.820 Hz
FIDRES 0.244532 Hz
AQ 4.0894465 sec
RG 49.98
DW 62.400 usec
DE 6.50 usec
TE 298.1 K
D1 1.00000000 sec
TD0 1
SFO1 400.1324710 MHz
NUC1 1H
P1 10.00 usec
PLW1 16.43099976 W

F2 - Processing parameters
SI 65536
SF 400.1300107 MHz
WDW EM
SSB 0
LB 0.30 Hz
GB 0
PC 1.00

ANL-MCL5-NMR-001

C5559-024-xn-2

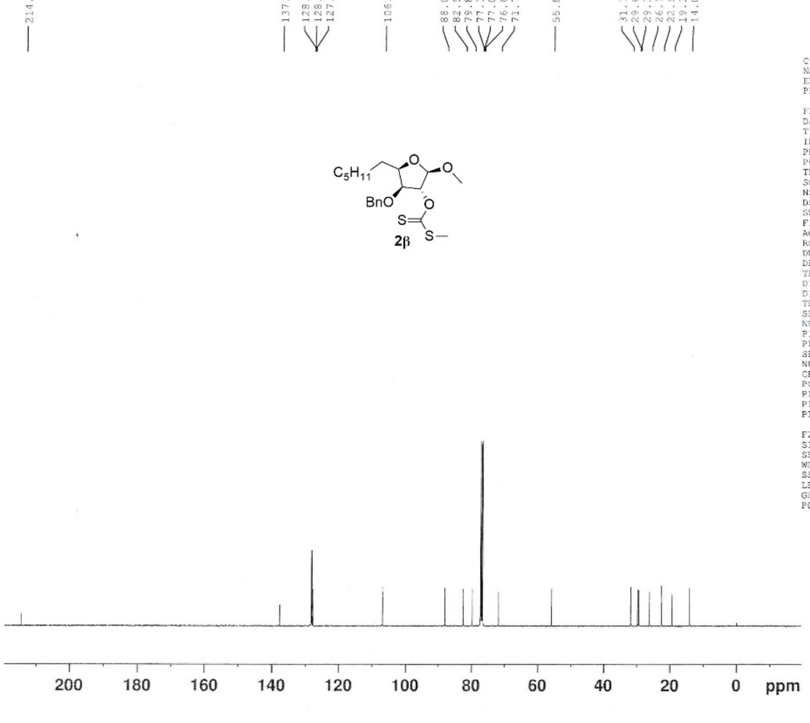

Current Data Parameters
NAME 512109D9034-C5559-024-xn-2
EXPNO 2
PROCNO 1

F2 - Acquisition Parameters
Date_ 20211003
Time 2.22 h
INSTRUM spect
PROBHD Z116098_0317 (
PULPROG zgpg30
TD 65536
SOLVENT CDCl3
NS 1024
DS 4
SWH 24038.461 Hz
FIDRES 0.733596 Hz
AQ 1.3631488 sec
RG 195.29
DW 20.800 usec
DE 6.50 usec
TE 298.2 K
D1 3.00000000 sec
D11 0.03000000 sec
TD0 1
SFO1 100.6228298 MHz
NUC1 13C
P1 10.00 usec
PLW1 77.83999634 W
SFO2 400.1316005 MHz
NUC2 1H
CPDPRG[2 waltz16
PCPD2 90.00 usec
PLW2 16.43099976 W
PLW12 0.20286000 W
PLW13 0.10204000 W

F2 - Processing parameters
SI 32768
SF 100.6127721 MHz
WDW EM
SSB 0
LB 2.00 Hz
GB 0
PC 1.40

Aragen Life Sciences Private Limited
Analytical - Discovery Chemistry

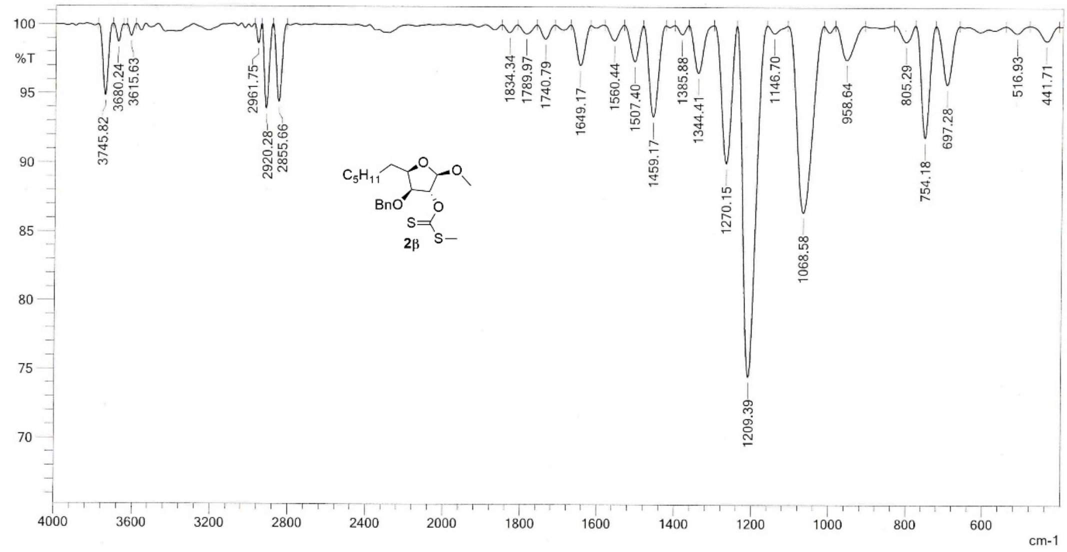

Sample Code : C5559-024-XN-2

Date : 20-Sep-2021 07:28:26 PM

Sample ID : 512109D8023

Instrument ID : ANL-MCL1-FT-IR-002

1

C5559-025-SN-1

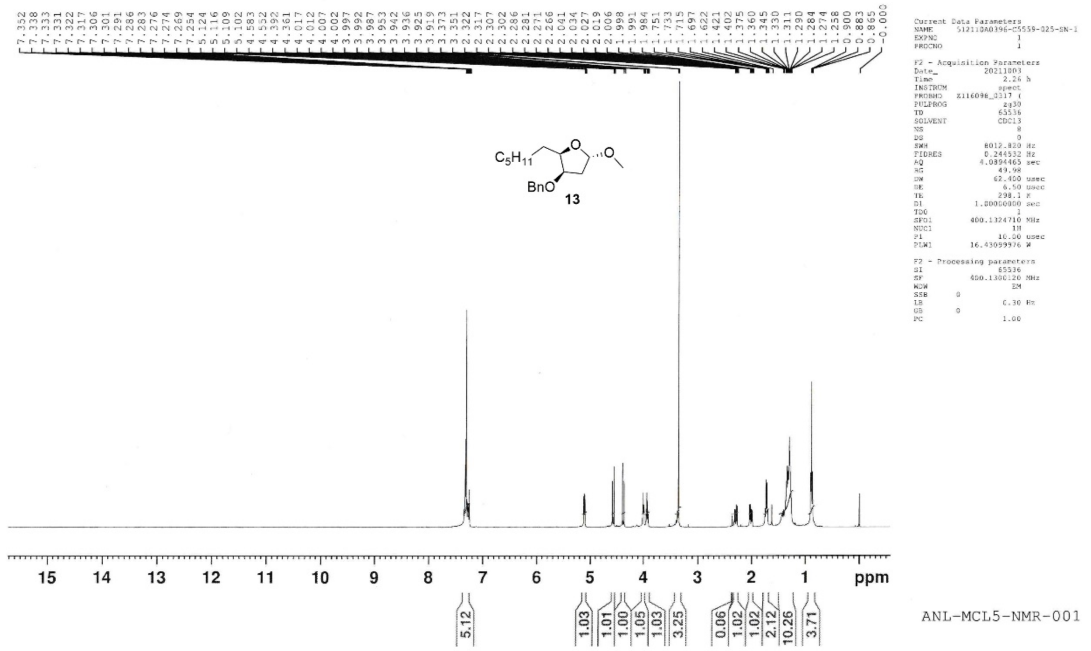

ANL-MCL5-NMR-001

C5559-025-SN-1

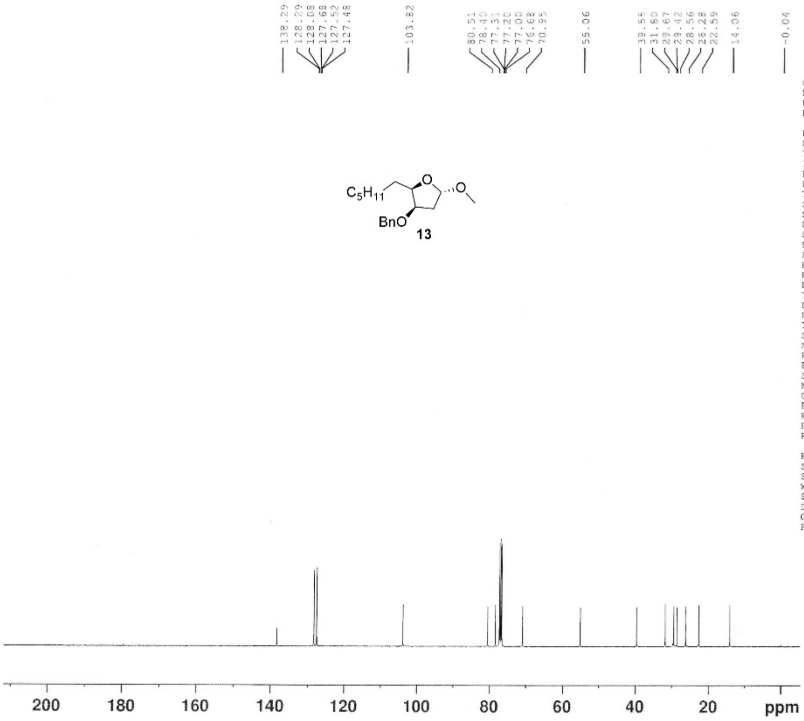


```
Current Data Parameters
NAME      512110A0396-C5559-025-SN-1
EXPNO                2
PROCNO               1

F2 - Acquisition Parameters
Date_          20211003
Time               3.43 h
INSTRUM           spect
PROBHD    Z116098_0317 (
PULPROG          zgpg30
TD                65536
SOLVENT           CDCl3
NS                 1024
DS                    4
SWH           24038.461 Hz
FIDRES         0.733596 Hz
AQ            1.3631488 sec
RG               195.29
DW               20.800 usec
DE                 6.50 usec
TE                298.2 K
D1           3.00000000 sec
D11          0.03000000 sec
TD0                   1
SFO1        100.6228298 MHz
NUC1                13C
P1                10.00 usec
PLW1        77.83999634 W
SFO2        400.1316005 MHz
NUC2                 1H
CPDPRG[2        waltz16
PCPD2             90.00 usec
PLW2        16.43099976 W
PLW12        0.20286000 W
PLW13        0.10204000 W

F2 - Processing parameters
SI                32768
SF          100.6127725 MHz
WDW                  EM
SSB      0
LB                 2.00 Hz
GB       0
PC                 1.40
```

aragen

Aragen Life Sciences Private Limited
Analytical - Discovery Chemistry

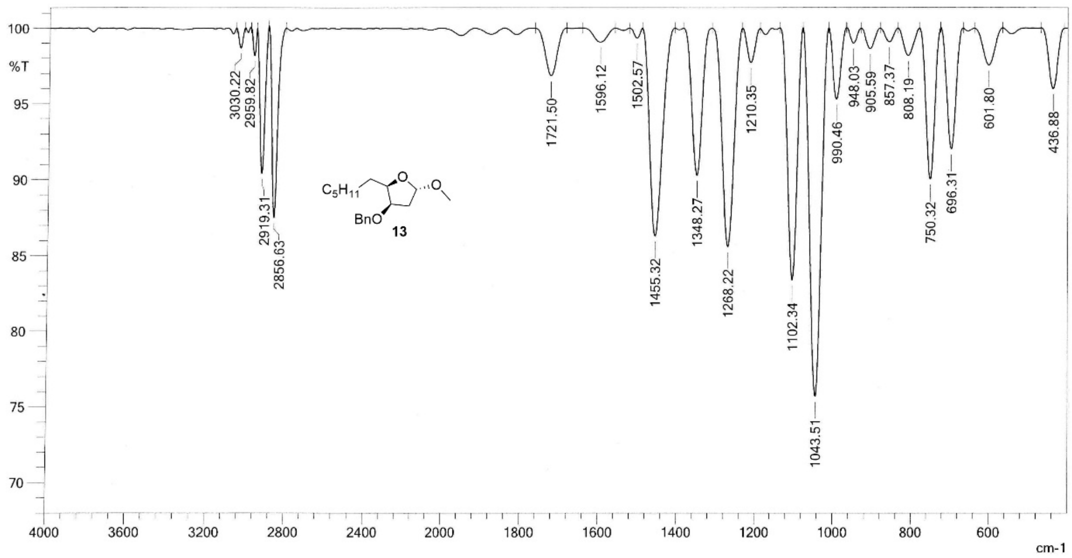

Sample Name : C5559-025-SN-1

Date : 01-Apr-2022 05:47:34 PM

Sample ID : 512204A0606

Instrument ID : ANL-MCL1-FT-IR-002

C5559-025-SN-2

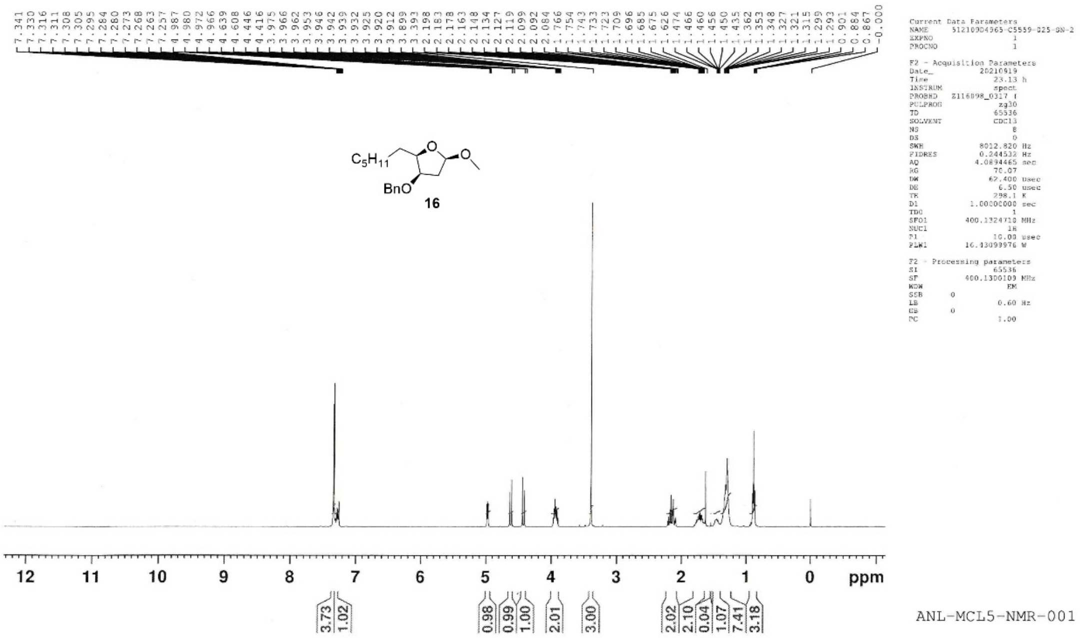

C5559-025-SN-2

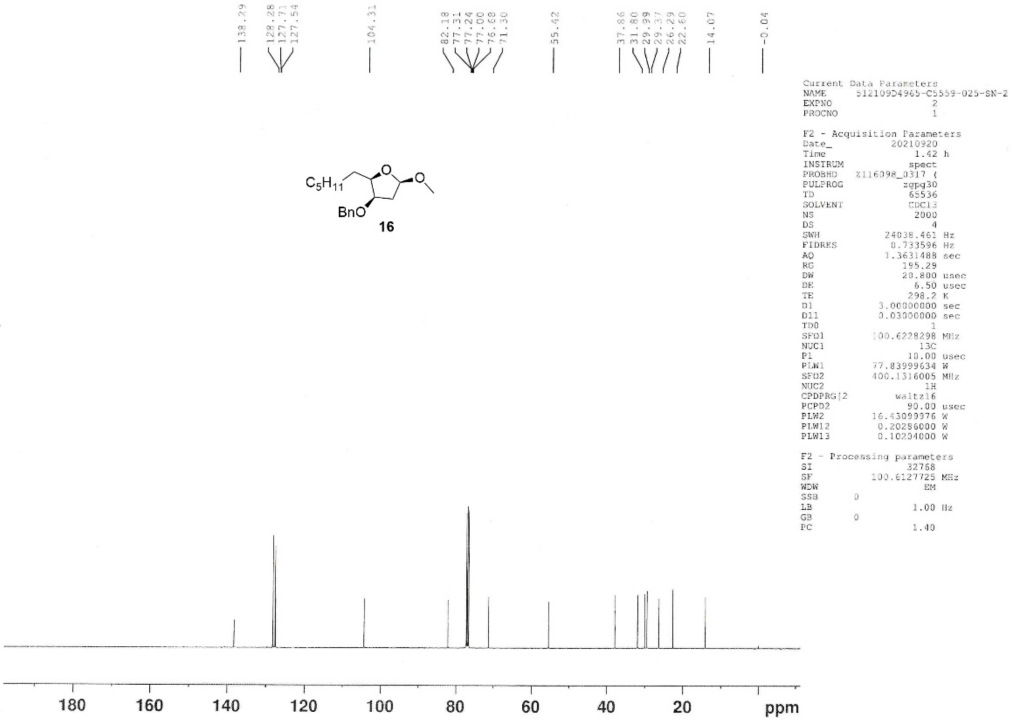

Aragen Life Sciences Private Limited
Analytical - Discovery Chemistry

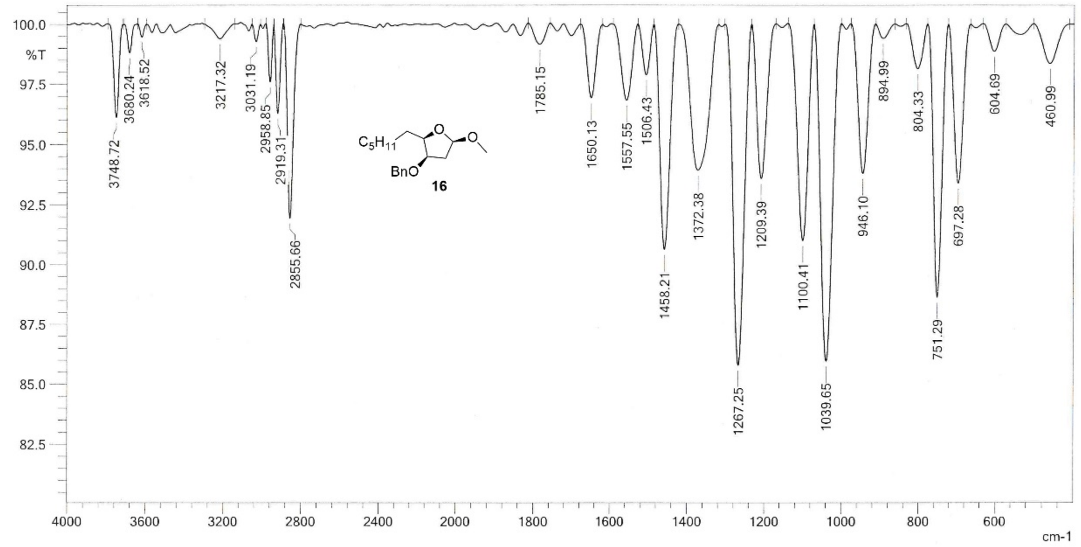

Sample Code : C5559-025-SN-2

Sample ID : 512109D8024

Date : 20-Sep-2021 07:19:25 PM

Instrument ID : ANL-MCL1-FT-IR-002

C5559-026-is-2

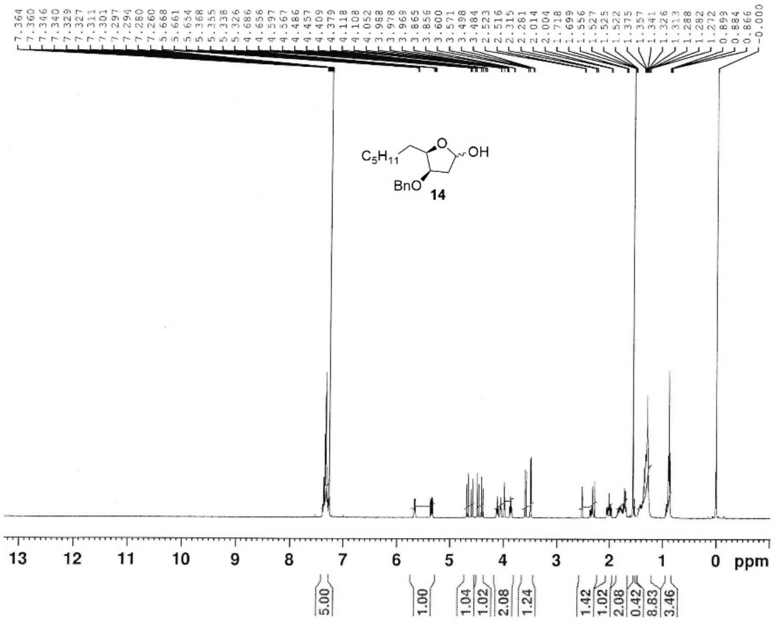


```
Current Data Parameters
NAME          512112G6111-C5559-026-is-2
EXPNO                    1
PROCNO                   1

F2 - Acquisition Parameters
Date_             20211229
Time                  6.43 h
INSTRUM  Avance Neo Nanobay 400
PROBHD    Z163739_0439 (
PULPROG              zg30
TD                  65536
SOLVENT             CDCl3
NS                      8
DS                      0
SWH              8196.722 Hz
FIDRES           0.250144 Hz
AQ              3.9976959 sec
RG                    101
DW                 61.000 usec
DE                  13.89 usec
TE                  298.1 K
D1             1.00000000 sec
TD0                     1
SFO1          400.2324714 MHz
NUC1                   1H
P0                   2.67 usec
P1                   8.00 usec
PLW1          22.80999947 W

F2 - Processing parameters
SI                  65536
SF            400.2300101 MHz
WDW                    EM
SSB                     0
LB                   0.30 Hz
GB                      0
PC                   1.00
```

ANL-MCL5-NMR-007

C5559-026-DP-2

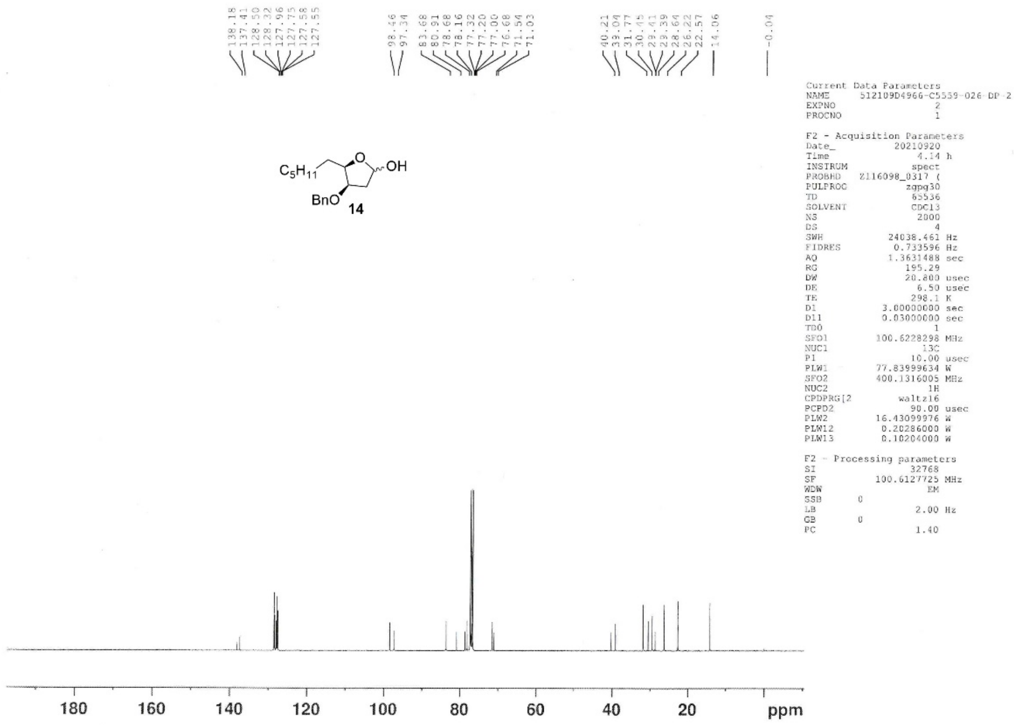


```
Current Data Parameters
NAME      512109D4966-C5559-026-DP-2
EXPNO                     2
PROCNO                    1

F2 - Acquisition Parameters
Date_             20210920
Time                  4.14 h
INSTRUM              spect
PROBHD    Z116098_0317 (
PULPROG             zgpg30
TD                   65536
SOLVENT              CDCl3
NS                    2000
DS                       4
SWH              24038.461 Hz
FIDRES            0.733596 Hz
AQ               1.3631488 sec
RG                  195.29
DW                  20.800 usec
DE                    6.50 usec
TE                   298.1 K
D1              3.00000000 sec
D11             0.03000000 sec
TD0                      1
SFO1           100.6228298 MHz
NUC1                   13C
P1                   10.00 usec
PLW1           77.83999634 W
SFO2           400.1316005 MHz
NUC2                    1H
CPDPRG[2           waltz16
PCPD2                90.00 usec
PLW2           16.43099976 W
PLW12           0.20286000 W
PLW13           0.10204000 W

F2 - Processing parameters
SI                   32768
SF             100.6127725 MHz
WDW                     EM
SSB       0
LB                    2.00 Hz
GB        0
PC                    1.40
```

aragen

Aragen Life Sciences Private Limited

Analytical - Discovery Chemistry

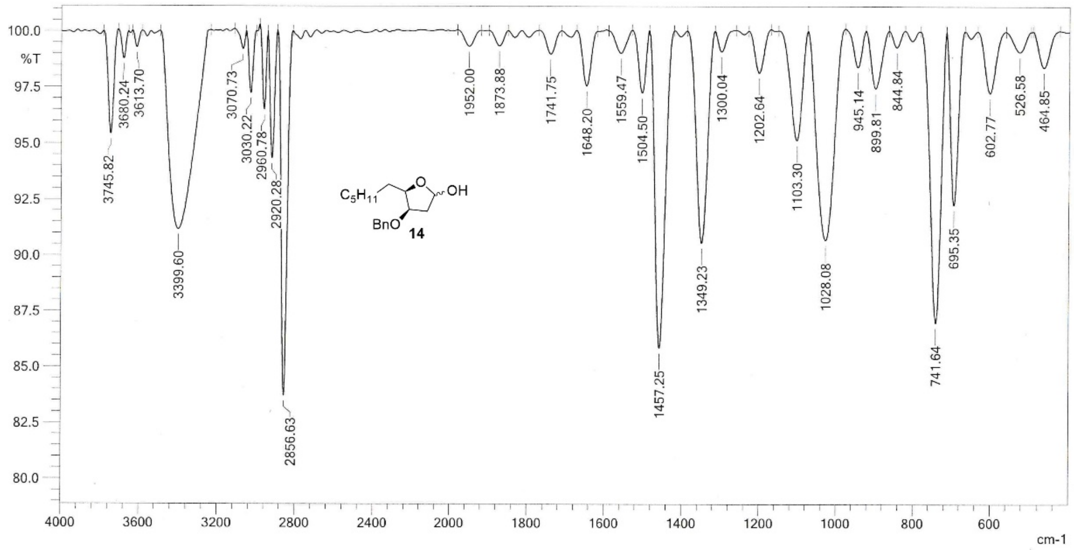

Sample Code : C5559-026-DP-2

Date : 20-Sep-2021 07:41:59 PM

Sample ID : 512109D8025

Instrument ID : ANL-MCL1-FT-IR-002

C5559-027-0X-2

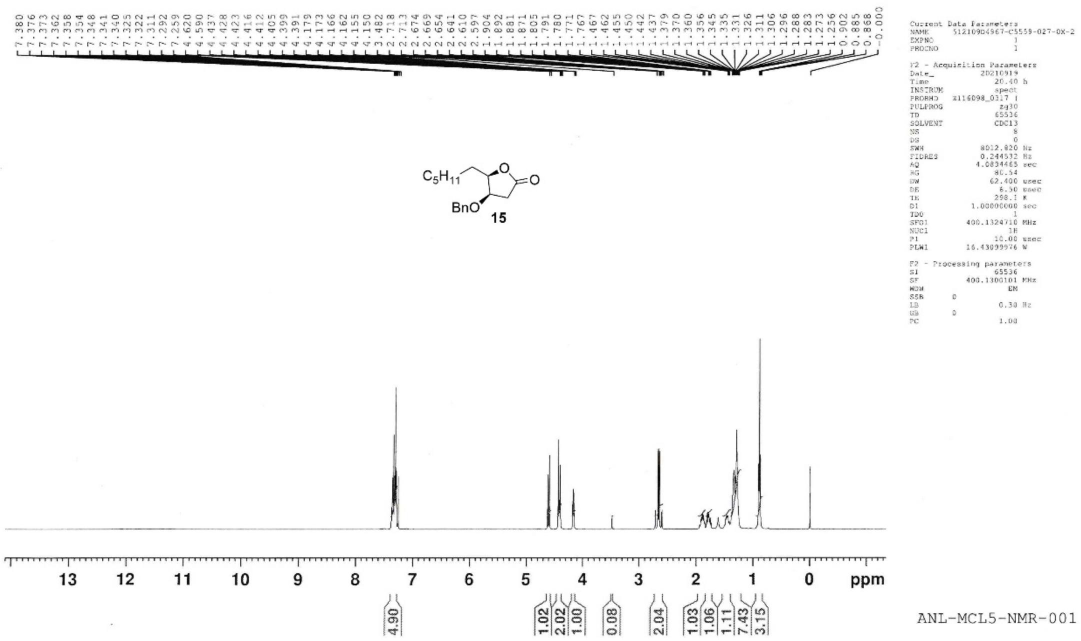

ANL-MCL5-NMR-001

C5559-027-0X-2

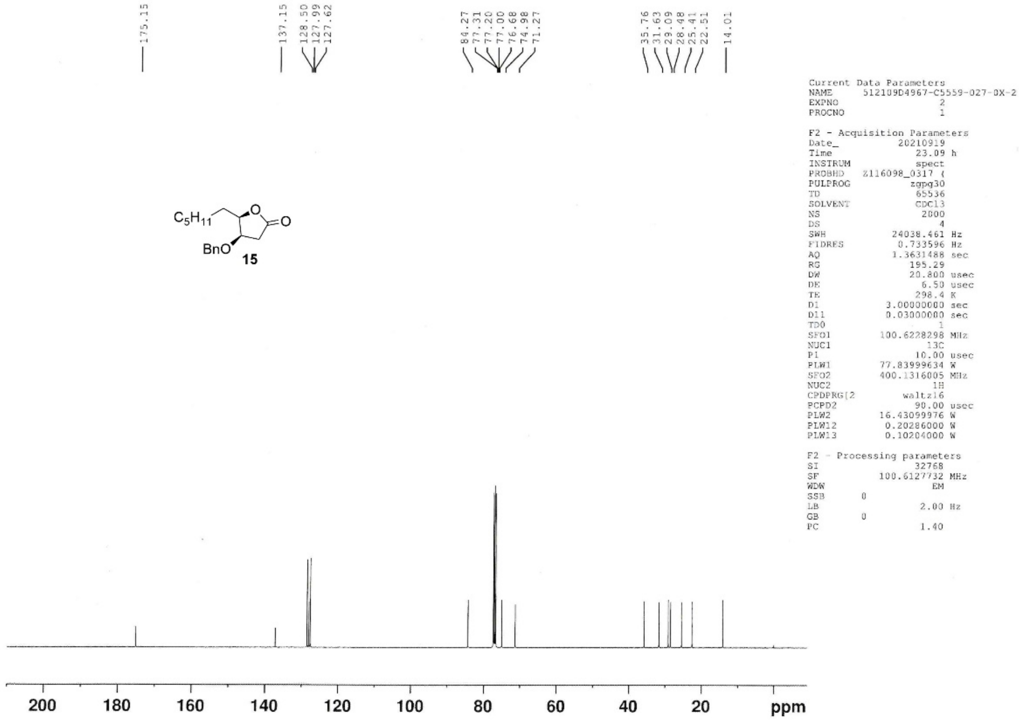

Aragen Life Sciences Private Limited
Analytical - Discovery Chemistry

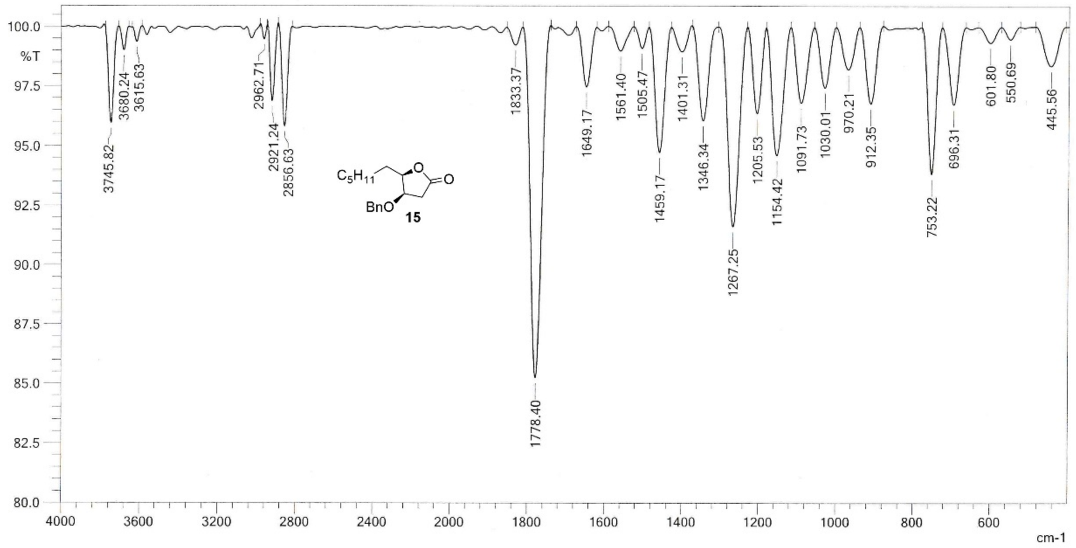

Sample Code : C5559-027-OX-2

Date : 20-Sep-2021 07:37:19 PM

Sample ID : 512109D8026

Instrument ID : ANL-MCL1-FT-IR-002

C5559-028-F

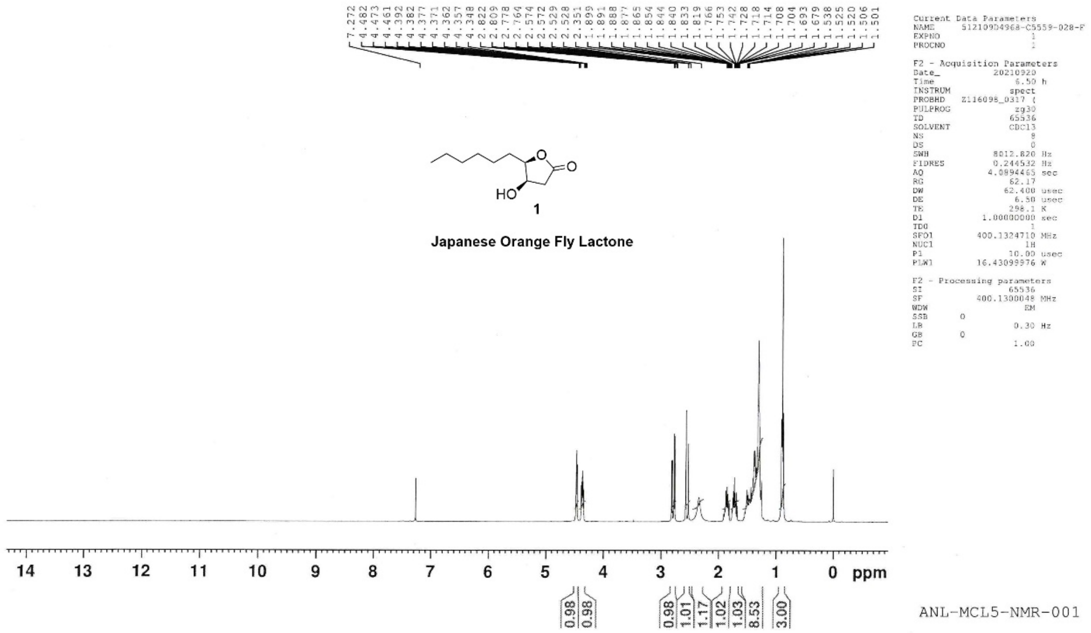

ANL-MCL5-NMR-001

C5559-028-F

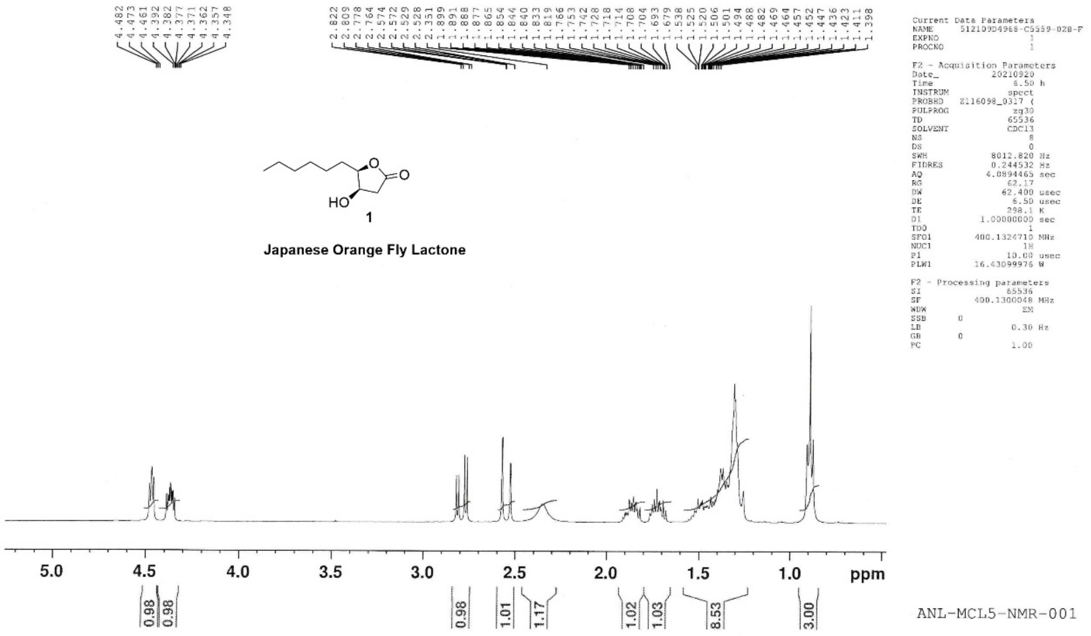

ANL-MCL5-NMR-001

C5559-028-F

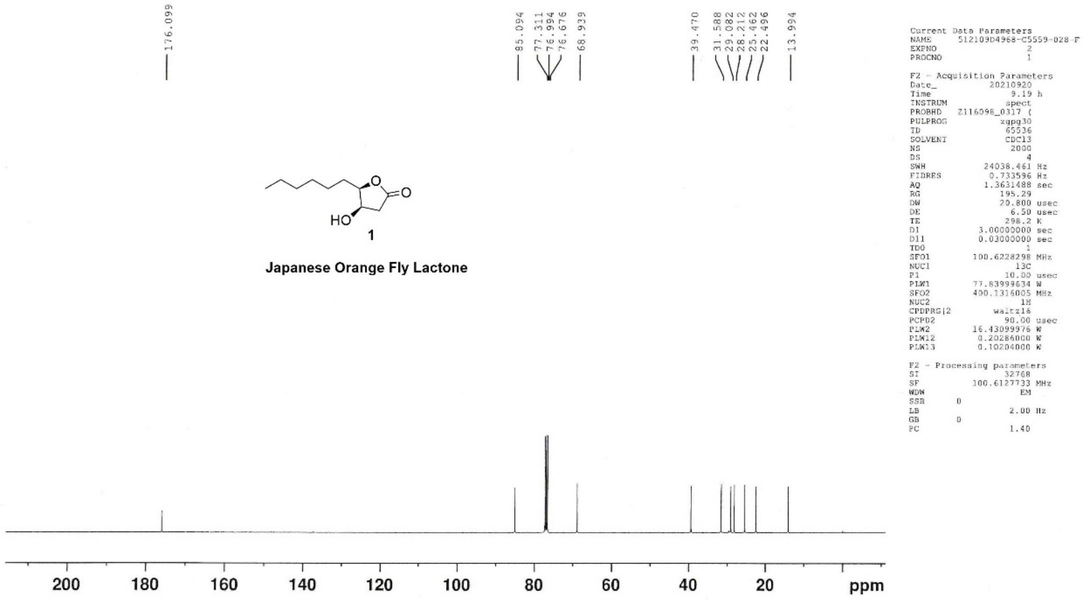

ANL-MCL5-NMR-001

Aragen Life Sciences Private Limited
Analytical - Discovery Chemistry

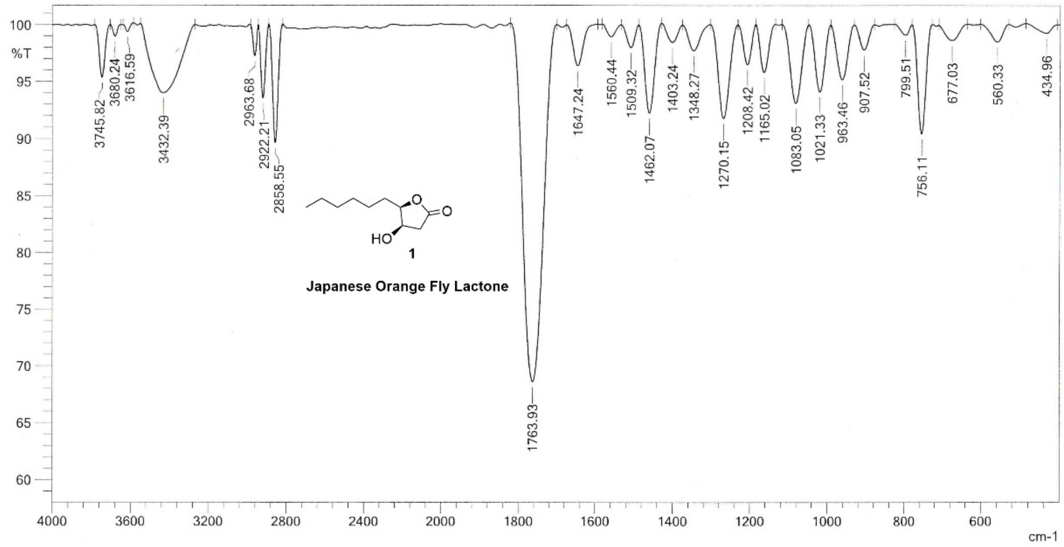

Sample Code : C5559-028-F

Date : 20-Sep-2021 07:23:56 PM

Sample ID : 512109D8027

Instrument ID : ANL-MCL1-FT-IR-002
